# Measurement of electroweak production of two jets in association with a Z boson in proton–proton collisions at $\sqrt{s}=8\,\text {TeV}$

**DOI:** 10.1140/epjc/s10052-014-3232-5

**Published:** 2015-02-10

**Authors:** V. Khachatryan, A. M. Sirunyan, A. Tumasyan, W. Adam, T. Bergauer, M. Dragicevic, J. Erö, C. Fabjan, M. Friedl, R. Frühwirth, V. M. Ghete, C. Hartl, N. Hörmann, J. Hrubec, M. Jeitler, W. Kiesenhofer, V. Knünz, M. Krammer, I. Krätschmer, D. Liko, I. Mikulec, D. Rabady, B. Rahbaran, H. Rohringer, R. Schöfbeck, J. Strauss, A. Taurok, W. Treberer-Treberspurg, W. Waltenberger, C.-E. Wulz, V. Mossolov, N. Shumeiko, J. Suarez Gonzalez, S. Alderweireldt, M. Bansal, S. Bansal, T. Cornelis, E. A. De Wolf, X. Janssen, A. Knutsson, S. Luyckx, S. Ochesanu, B. Roland, R. Rougny, M. Van De Klundert, H. Van Haevermaet, P. Van Mechelen, N. Van Remortel, A. Van Spilbeeck, F. Blekman, S. Blyweert, J. D’Hondt, N. Daci, N. Heracleous, J. Keaveney, S. Lowette, M. Maes, A. Olbrechts, Q. Python, D. Strom, S. Tavernier, W. Van Doninck, P. Van Mulders, G. P. Van Onsem, I. Villella, C. Caillol, B. Clerbaux, G. De Lentdecker, D. Dobur, L. Favart, A. P. R. Gay, A. Grebenyuk, A. Léonard, A. Mohammadi, L. Perniè, T. Reis, T. Seva, L. Thomas, C. Vander Velde, P. Vanlaer, J. Wang, V. Adler, K. Beernaert, L. Benucci, A. Cimmino, S. Costantini, S. Crucy, S. Dildick, A. Fagot, G. Garcia, J. Mccartin, A. A. Ocampo Rios, D. Ryckbosch, S. Salva Diblen, M. Sigamani, N. Strobbe, F. Thyssen, M. Tytgat, E. Yazgan, N. Zaganidis, S. Basegmez, C. Beluffi, G. Bruno, R. Castello, A. Caudron, L. Ceard, G. G. Da Silveira, C. Delaere, T. du Pree, D. Favart, L. Forthomme, A. Giammanco, J. Hollar, P. Jez, M. Komm, V. Lemaitre, C. Nuttens, D. Pagano, L. Perrini, A. Pin, K. Piotrzkowski, A. Popov, L. Quertenmont, M. Selvaggi, M. Vidal Marono, J. M. Vizan Garcia, N. Beliy, T. Caebergs, E. Daubie, G. H. Hammad, W. L. Aldá Júnior, G. A. Alves, L. Brito, M. Correa Martins Junior, T. Dos Reis Martins, C. Mora Herrera, M. E. Pol, W. Carvalho, J. Chinellato, A. Custódio, E. M. Da Costa, D. De Jesus Damiao, C. De Oliveira Martins, S. Fonseca De Souza, H. Malbouisson, D. Matos Figueiredo, L. Mundim, H. Nogima, W. L. Prado Da Silva, J. Santaolalla, A. Santoro, A. Sznajder, E. J. Tonelli Manganote, A. Vilela Pereira, C. A. Bernardes, S. Dogra, T. R. Fernandez Perez Tomei, E. M. Gregores, P. G. Mercadante, S. F. Novaes, Sandra S. Padula, A. Aleksandrov, V. Genchev, P. Iaydjiev, A. Marinov, S. Piperov, M. Rodozov, S. Stoykova, G. Sultanov, V. Tcholakov, M. Vutova, A. Dimitrov, I. Glushkov, R. Hadjiiska, V. Kozhuharov, L. Litov, B. Pavlov, P. Petkov, J. G. Bian, G. M. Chen, H. S. Chen, M. Chen, R. Du, C. H. Jiang, S. Liang, R. Plestina, J. Tao, X. Wang, Z. Wang, C. Asawatangtrakuldee, Y. Ban, Y. Guo, Q. Li, W. Li, S. Liu, Y. Mao, S. J. Qian, D. Wang, L. Zhang, W. Zou, C. Avila, L. F. Chaparro Sierra, C. Florez, J. P. Gomez, B. Gomez Moreno, J. C. Sanabria, N. Godinovic, D. Lelas, D. Polic, I. Puljak, Z. Antunovic, M. Kovac, V. Brigljevic, K. Kadija, J. Luetic, D. Mekterovic, L. Sudic, A. Attikis, G. Mavromanolakis, J. Mousa, C. Nicolaou, F. Ptochos, P. A. Razis, M. Bodlak, M. Finger, M. Finger, Y. Assran, A. Ellithi Kamel, M. A. Mahmoud, A. Radi, M. Kadastik, M. Murumaa, M. Raidal, A. Tiko, P. Eerola, G. Fedi, M. Voutilainen, J. Härkönen, V. Karimäki, R. Kinnunen, M. J. Kortelainen, T. Lampén, K. Lassila-Perini, S. Lehti, T. Lindén, P. Luukka, T. Mäenpää, T. Peltola, E. Tuominen, J. Tuominiemi, E. Tuovinen, L. Wendland, J. Talvitie, T. Tuuva, M. Besancon, F. Couderc, M. Dejardin, D. Denegri, B. Fabbro, J. L. Faure, C. Favaro, F. Ferri, S. Ganjour, A. Givernaud, P. Gras, G. Hamel de Monchenault, P. Jarry, E. Locci, J. Malcles, J. Rander, A. Rosowsky, M. Titov, S. Baffioni, F. Beaudette, P. Busson, C. Charlot, T. Dahms, M. Dalchenko, L. Dobrzynski, N. Filipovic, A. Florent, R. Granier de Cassagnac, L. Mastrolorenzo, P. Miné, C. Mironov, I. N. Naranjo, M. Nguyen, C. Ochando, P. Paganini, S. Regnard, R. Salerno, J. B. Sauvan, Y. Sirois, C. Veelken, Y. Yilmaz, A. Zabi, J.-L. Agram, J. Andrea, A. Aubin, D. Bloch, J.-M. Brom, E. C. Chabert, C. Collard, E. Conte, J.-C. Fontaine, D. Gelé, U. Goerlach, C. Goetzmann, A.-C. Le Bihan, P. Van Hove, S. Gadrat, S. Beauceron, N. Beaupere, G. Boudoul, E. Bouvier, S. Brochet, C. A. Carrillo Montoya, J. Chasserat, R. Chierici, D. Contardo, P. Depasse, H. El Mamouni, J. Fan, J. Fay, S. Gascon, M. Gouzevitch, B. Ille, T. Kurca, M. Lethuillier, L. Mirabito, S. Perries, J. D. Ruiz Alvarez, D. Sabes, L. Sgandurra, V. Sordini, M. Vander Donckt, P. Verdier, S. Viret, H. Xiao, Z. Tsamalaidze, C. Autermann, S. Beranek, M. Bontenackels, M. Edelhoff, L. Feld, O. Hindrichs, K. Klein, A. Ostapchuk, A. Perieanu, F. Raupach, J. Sammet, S. Schael, H. Weber, B. Wittmer, V. Zhukov, M. Ata, M. Brodski, E. Dietz-Laursonn, D. Duchardt, M. Erdmann, R. Fischer, A. Güth, T. Hebbeker, C. Heidemann, K. Hoepfner, D. Klingebiel, S. Knutzen, P. Kreuzer, M. Merschmeyer, A. Meyer, P. Millet, M. Olschewski, K. Padeken, P. Papacz, H. Reithler, S. A. Schmitz, L. Sonnenschein, D. Teyssier, S. Thüer, M. Weber, V. Cherepanov, Y. Erdogan, G. Flügge, H. Geenen, M. Geisler, W. Haj Ahmad, A. Heister, F. Hoehle, B. Kargoll, T. Kress, Y. Kuessel, J. Lingemann, A. Nowack, I. M. Nugent, L. Perchalla, O. Pooth, A. Stahl, I. Asin, N. Bartosik, J. Behr, W. Behrenhoff, U. Behrens, A. J. Bell, M. Bergholz, A. Bethani, K. Borras, A. Burgmeier, A. Cakir, L. Calligaris, A. Campbell, S. Choudhury, F. Costanza, C. Diez Pardos, S. Dooling, T. Dorland, G. Eckerlin, D. Eckstein, T. Eichhorn, G. Flucke, J. Garay Garcia, A. Geiser, P. Gunnellini, J. Hauk, M. Hempel, D. Horton, H. Jung, A. Kalogeropoulos, M. Kasemann, P. Katsas, J. Kieseler, C. Kleinwort, D. Krücker, W. Lange, J. Leonard, K. Lipka, A. Lobanov, W. Lohmann, B. Lutz, R. Mankel, I. Marfin, I.-A. Melzer-Pellmann, A. B. Meyer, G. Mitta, J. Mnich, A. Mussgiller, S. Naumann-Emme, A. Nayak, O. Novgorodova, F. Nowak, E. Ntomari, H. Perrey, D. Pitzl, R. Placakyte, A. Raspereza, P. M. Ribeiro Cipriano, E. Ron, M. Ö. Sahin, J. Salfeld-Nebgen, P. Saxena, R. Schmidt, T. Schoerner-Sadenius, M. Schröder, C. Seitz, S. Spannagel, A. D. R. Vargas Trevino, R. Walsh, C. Wissing, M. Aldaya Martin, V. Blobel, M. Centis Vignali, A. R. Draeger, J. Erfle, E. Garutti, K. Goebel, M. Görner, J. Haller, M. Hoffmann, R. S. Höing, H. Kirschenmann, R. Klanner, R. Kogler, J. Lange, T. Lapsien, T. Lenz, I. Marchesini, J. Ott, T. Peiffer, N. Pietsch, J. Poehlsen, T. Poehlsen, D. Rathjens, C. Sander, H. Schettler, P. Schleper, E. Schlieckau, A. Schmidt, M. Seidel, V. Sola, H. Stadie, G. Steinbrück, D. Troendle, E. Usai, L. Vanelderen, C. Barth, C. Baus, J. Berger, C. Böser, E. Butz, T. Chwalek, W. De Boer, A. Descroix, A. Dierlamm, M. Feindt, F. Frensch, M. Giffels, F. Hartmann, T. Hauth, U. Husemann, I. Katkov, A. Kornmayer, E. Kuznetsova, P. Lobelle Pardo, M. U. Mozer, Th. Müller, A. Nürnberg, G. Quast, K. Rabbertz, F. Ratnikov, S. Röcker, H. J. Simonis, F. M. Stober, R. Ulrich, J. Wagner-Kuhr, S. Wayand, T. Weiler, R. Wolf, G. Anagnostou, G. Daskalakis, T. Geralis, V. A. Giakoumopoulou, A. Kyriakis, D. Loukas, A. Markou, C. Markou, A. Psallidas, I. Topsis-Giotis, A. Panagiotou, A. Agapitos, S. Kesisoglou, N. Saoulidou, E. Stiliaris, X. Aslanoglou, I. Evangelou, G. Flouris, C. Foudas, P. Kokkas, N. Manthos, I. Papadopoulos, E. Paradas, G. Bencze, C. Hajdu, P. Hidas, D. Horvath, F. Sikler, V. Veszpremi, G. Vesztergombi, A. J. Zsigmond, N. Beni, S. Czellar, J. Karancsi, J. Molnar, J. Palinkas, Z. Szillasi, P. Raics, Z. L. Trocsanyi, B. Ujvari, S. K. Swain, S. B. Beri, V. Bhatnagar, R. Gupta, U. Bhawandeep, A. K. Kalsi, M. Kaur, M. Mittal, N. Nishu, J. B. Singh, Ashok Kumar, Arun Kumar, S. Ahuja, A. Bhardwaj, B. C. Choudhary, A. Kumar, S. Malhotra, M. Naimuddin, K. Ranjan, V. Sharma, S. Banerjee, S. Bhattacharya, K. Chatterjee, S. Dutta, B. Gomber, Sa. Jain, Sh. Jain, R. Khurana, A. Modak, S. Mukherjee, D. Roy, S. Sarkar, M. Sharan, A. Abdulsalam, D. Dutta, S. Kailas, V. Kumar, A. K. Mohanty, L. M. Pant, P. Shukla, A. Topkar, T. Aziz, S. Banerjee, S. Bhowmik, R. M. Chatterjee, R. K. Dewanjee, S. Dugad, S. Ganguly, S. Ghosh, M. Guchait, A. Gurtu, G. Kole, S. Kumar, M. Maity, G. Majumder, K. Mazumdar, G. B. Mohanty, B. Parida, K. Sudhakar, N. Wickramage, H. Bakhshiansohi, H. Behnamian, S. M. Etesami, A. Fahim, R. Goldouzian, A. Jafari, M. Khakzad, M. Mohammadi Najafabadi, M. Naseri, S. Paktinat Mehdiabadi, F Rezaei Hosseinabadi, B. Safarzadeh, M. Zeinali, M. Felcini, M. Grunewald, M. Abbrescia, L. Barbone, C. Calabria, S. S. Chhibra, A. Colaleo, D. Creanza, N. De Filippis, M. De Palma, L. Fiore, G. Iaselli, G. Maggi, M. Maggi, S. My, S. Nuzzo, A. Pompili, G. Pugliese, R. Radogna, G. Selvaggi, L. Silvestris, G. Singh, R. Venditti, P. Verwilligen, G. Zito, G. Abbiendi, A. C. Benvenuti, D. Bonacorsi, S. Braibant-Giacomelli, L. Brigliadori, R. Campanini, P. Capiluppi, A. Castro, F. R. Cavallo, G. Codispoti, M. Cuffiani, G. M. Dallavalle, F. Fabbri, A. Fanfani, D. Fasanella, P. Giacomelli, C. Grandi, L. Guiducci, S. Marcellini, G. Masetti, A. Montanari, F. L. Navarria, A. Perrotta, F. Primavera, A. M. Rossi, T. Rovelli, G. P. Siroli, N. Tosi, R. Travaglini, S. Albergo, G. Cappello, M. Chiorboli, S. Costa, F. Giordano, R. Potenza, A. Tricomi, C. Tuve, G. Barbagli, V. Ciulli, C. Civinini, R. D’Alessandro, E. Focardi, E. Gallo, S. Gonzi, V. Gori, P. Lenzi, M. Meschini, S. Paoletti, G. Sguazzoni, A. Tropiano, L. Benussi, S. Bianco, F. Fabbri, D. Piccolo, F. Ferro, M. Lo Vetere, E. Robutti, S. Tosi, M. E. Dinardo, S. Fiorendi, S. Gennai, R. Gerosa, A. Ghezzi, P. Govoni, M. T. Lucchini, S. Malvezzi, R. A. Manzoni, A. Martelli, B. Marzocchi, D. Menasce, L. Moroni, M. Paganoni, D. Pedrini, S. Ragazzi, N. Redaelli, T. Tabarelli de Fatis, S. Buontempo, N. Cavallo, S. Di Guida, F. Fabozzi, A. O. M. Iorio, L. Lista, S. Meola, M. Merola, P. Paolucci, P. Azzi, N. Bacchetta, M. Bellato, M. Biasotto, A. Branca, M. Dall’Osso, T. Dorigo, U. Dosselli, M. Galanti, F. Gasparini, P. Giubilato, A. Gozzelino, K. Kanishchev, S. Lacaprara, M. Margoni, A. T. Meneguzzo, J. Pazzini, N. Pozzobon, P. Ronchese, F. Simonetto, E. Torassa, M. Tosi, A. Trioss, S. Vanini, S. Ventura, P. Zotto, A. Zucchetta, M. Gabusi, S. P. Ratti, C. Riccardi, P. Salvini, P. Vitulo, M. Biasini, G. M. Bilei, D. Ciangottini, L. Fanò, P. Lariccia, G. Mantovani, M. Menichelli, F. Romeo, A. Saha, A. Santocchia, A. Spiezia, K. Androsov, P. Azzurri, G. Bagliesi, J. Bernardini, T. Boccali, G. Broccolo, D. Caiulo, R. Castaldi, M. A. Ciocci, R. Dell’Orso, S. Donato, F. Fiori, L. Foà, A. Giassi, M. T. Grippo, F. Ligabue, T. Lomtadze, L. Martini, A. Messineo, C. S. Moon, F. Palla, A. Rizzi, A. Savoy-Navarro, A. T. Serban, P. Spagnolo, P. Squillacioti, R. Tenchini, G. Tonelli, A. Venturi, P. G. Verdini, C. Vernieri, L. Barone, F. Cavallari, G. D’imperio, D. Del Re, M. Diemoz, M. Grassi, C. Jorda, E. Longo, F. Margaroli, P. Meridiani, F. Micheli, S. Nourbakhsh, G. Organtini, R. Paramatti, S. Rahatlou, C. Rovelli, F. Santanastasio, L. Soffi, P. Traczyk, N. Amapane, R. Arcidiacono, S. Argiro, M. Arneodo, R. Bellan, C. Biino, N. Cartiglia, S. Casasso, M. Costa, A. Degano, N. Demaria, L. Finco, C. Mariotti, S. Maselli, E. Migliore, V. Monaco, M. Musich, M. M. Obertino, G. Ortona, L. Pacher, N. Pastrone, M. Pelliccioni, G. L. Pinna Angioni, A. Potenza, A. Romero, M. Ruspa, R. Sacchi, A. Solano, A. Staiano, U. Tamponi, S. Belforte, V. Candelise, M. Casarsa, F. Cossutti, G. Della Ricca, B. Gobbo, C. La Licata, M. Marone, D. Montanino, A. Schizzi, T. Umer, A. Zanetti, S. Chang, A. Kropivnitskaya, S. K. Nam, D. H. Kim, G. N. Kim, M. S. Kim, D. J. Kong, S. Lee, Y. D. Oh, H. Park, A. Sakharov, D. C. Son, T. J. Kim, J. Y. Kim, S. Song, S. Choi, D. Gyun, B. Hong, M. Jo, H. Kim, Y. Kim, B. Lee, K. S. Lee, S. K. Park, Y. Roh, M. Choi, J. H. Kim, I. C. Park, S. Park, G. Ryu, M. S. Ryu, Y. Choi, Y. K. Choi, J. Goh, D. Kim, E. Kwon, J. Lee, H. Seo, I. Yu, A. Juodagalvis, J. R. Komaragiri, M. A B. Md Ali, H. Castilla-Valdez, E. De La Cruz-Burelo, I. Heredia-de La Cruz, R. Lopez-Fernandez, A. Sanchez-Hernandez, S. Carrillo Moreno, F. Vazquez Valencia, I. Pedraza, H. A. Salazar Ibarguen, E. Casimiro Linares, A. Morelos Pineda, D. Krofcheck, P. H. Butler, S. Reucroft, A. Ahmad, M. Ahmad, Q. Hassan, H. R. Hoorani, S. Khalid, W. A. Khan, T. Khurshid, M. A. Shah, M. Shoaib, H. Bialkowska, M. Bluj, B. Boimska, T. Frueboes, M. Górski, M. Kazana, K. Nawrocki, K. Romanowska-Rybinska, M. Szleper, P. Zalewski, G. Brona, K. Bunkowski, M. Cwiok, W. Dominik, K. Doroba, A. Kalinowski, M. Konecki, J. Krolikowski, M. Misiura, M. Olszewski, W. Wolszczak, P. Bargassa, C. Beirão Da Cruz E Silva, P. Faccioli, P. G. Ferreira Parracho, M. Gallinaro, F. Nguyen, J. Rodrigues Antunes, J. Seixas, J. Varela, P. Vischia, S. Afanasiev, P. Bunin, M. Gavrilenko, I. Golutvin, I. Gorbunov, A. Kamenev, V. Karjavin, V. Konoplyanikov, A. Lanev, A. Malakhov, V. Matveev, P. Moisenz, V. Palichik, V. Perelygin, S. Shmatov, N. Skatchkov, V. Smirnov, A. Zarubin, V. Golovtsov, Y. Ivanov, V. Kim, P. Levchenko, V. Murzin, V. Oreshkin, I. Smirnov, V. Sulimov, L. Uvarov, S. Vavilov, A. Vorobyev, An. Vorobyev, Yu. Andreev, A. Dermenev, S. Gninenko, N. Golubev, M. Kirsanov, N. Krasnikov, A. Pashenkov, D. Tlisov, A. Toropin, V. Epshteyn, V. Gavrilov, N. Lychkovskaya, V. Popov, G. Safronov, S. Semenov, A. Spiridonov, V. Stolin, E. Vlasov, A. Zhokin, V. Andreev, M. Azarkin, I. Dremin, M. Kirakosyan, A. Leonidov, G. Mesyats, S. V. Rusakov, A. Vinogradov, A. Belyaev, E. Boos, A. Ershov, A. Gribushin, L. Khein, V. Klyukhin, O. Kodolova, I. Lokhtin, O. Lukina, S. Obraztsov, S. Petrushanko, V. Savrin, A. Snigirev, I. Azhgirey, I. Bayshev, S. Bitioukov, V. Kachanov, A. Kalinin, D. Konstantinov, V. Krychkine, V. Petrov, R. Ryutin, A. Sobol, L. Tourtchanovitch, S. Troshin, N. Tyurin, A. Uzunian, A. Volkov, P. Adzic, M. Ekmedzic, J. Milosevic, V. Rekovic, J. Alcaraz Maestre, C. Battilana, E. Calvo, M. Cerrada, M. Chamizo Llatas, N. Colino, B. De La Cruz, A. Delgado Peris, D. Domínguez Vázquez, A. Escalante Del Valle, C. Fernandez Bedoya, J. P. Fernández Ramos, J. Flix, M. C. Fouz, P. Garcia-Abia, O. Gonzalez Lopez, S. Goy Lopez, J. M. Hernandez, M. I. Josa, G. Merino, E. Navarro De Martino, A. Pérez-Calero Yzquierdo, J. Puerta Pelayo, A. Quintario Olmeda, I. Redondo, L. Romero, M. S. Soares, C. Albajar, J. F. de Trocóniz, M. Missiroli, D. Moran, H. Brun, J. Cuevas, J. Fernandez Menendez, S. Folgueras, I. Gonzalez Caballero, L. Lloret Iglesias, J. A. Brochero Cifuentes, I. J. Cabrillo, A. Calderon, J. Duarte Campderros, M. Fernandez, G. Gomez, A. Graziano, A. Lopez Virto, J. Marco, R. Marco, C. Martinez Rivero, F. Matorras, F. J. Munoz Sanchez, J. Piedra Gomez, T. Rodrigo, A. Y. Rodríguez-Marrero, A. Ruiz-Jimeno, L. Scodellaro, I. Vila, R. Vilar Cortabitarte, D. Abbaneo, E. Auffray, G. Auzinger, M. Bachtis, P. Baillon, A. H. Ball, D. Barney, A. Benaglia, J. Bendavid, L. Benhabib, J. F. Benitez, C. Bernet, G. Bianchi, P. Bloch, A. Bocci, A. Bonato, O. Bondu, C. Botta, H. Breuker, T. Camporesi, G. Cerminara, S. Colafranceschi, M. D’Alfonso, D. d’Enterria, A. Dabrowski, A. David, F. De Guio, A. De Roeck, S. De Visscher, M. Dobson, M. Dordevic, N. Dupont-Sagorin, A. Elliott-Peisert, J. Eugster, G. Franzoni, W. Funk, D. Gigi, K. Gill, D. Giordano, M. Girone, F. Glege, R. Guida, S. Gundacker, M. Guthoff, R. Guida, J. Hammer, M. Hansen, P. Harris, J. Hegeman, V. Innocente, P. Janot, K. Kousouris, K. Krajczar, P. Lecoq, C. Lourenço, N. Magini, L. Malgeri, M. Mannelli, J. Marrouche, L. Masetti, F. Meijers, S. Mersi, E. Meschi, F. Moortgat, S. Morovic, M. Mulders, P. Musella, L. Orsini, L. Pape, E. Perez, L. Perrozzi, A. Petrilli, G. Petrucciani, A. Pfeiffer, M. Pierini, M. Pimiä, D. Piparo, M. Plagge, A. Racz, G. Rolandi, M. Rovere, H. Sakulin, C. Schäfer, C. Schwick, A. Sharma, P. Siegrist, P. Silva, M. Simon, P. Sphicas, D. Spiga, J. Steggemann, B. Stieger, M. Stoye, Y. Takahashi, D. Treille, A. Tsirou, G. I. Veres, J. R. Vlimant, N. Wardle, H. K. Wöhri, H. Wollny, W. D. Zeuner, W. Bertl, K. Deiters, W. Erdmann, R. Horisberger, Q. Ingram, H. C. Kaestli, D. Kotlinski, U. Langenegger, D. Renker, T. Rohe, F. Bachmair, L. Bäni, L. Bianchini, P. Bortignon, M. A. Buchmann, B. Casal, N. Chanon, A. Deisher, G. Dissertori, M. Dittmar, M. Donegà, M. Dünser, P. Eller, C. Grab, D. Hits, W. Lustermann, B. Mangano, A. C. Marini, P. Martinez Ruiz del Arbol, D. Meister, N. Mohr, C. Nägeli, F. Nessi-Tedaldi, F. Pandolfi, F. Pauss, M. Peruzzi, M. Quittnat, L. Rebane, M. Rossini, A. Starodumov, M. Takahashi, K. Theofilatos, R. Wallny, H. A. Weber, C. Amsler, M. F. Canelli, V. Chiochia, A. De Cosa, A. Hinzmann, T. Hreus, B. Kilminster, C Lange, B. Millan Mejias, J. Ngadiuba, P. Robmann, F. J. Ronga, S. Taroni, M. Verzetti, Y. Yang, M. Cardaci, K. H. Chen, C. Ferro, C. M. Kuo, W. Lin, Y. J. Lu, R. Volpe, S. S. Yu, P. Chang, Y. H. Chang, Y. W. Chang, Y. Chao, K. F. Chen, P. H. Chen, C. Dietz, U. Grundler, W.-S. Hou, K. Y. Kao, Y. J. Lei, Y. F. Liu, R.-S. Lu, D. Majumder, E. Petrakou, Y. M. Tzeng, R. Wilken, B. Asavapibhop, N. Srimanobhas, N. Suwonjandee, A. Adiguzel, M. N. Bakirci, S. Cerci, C. Dozen, I. Dumanoglu, E. Eskut, S. Girgis, G. Gokbulut, E. Gurpinar, I. Hos, E. E. Kangal, A. Kayis Topaksu, G. Onengut, K. Ozdemir, S. Ozturk, A. Polatoz, K. Sogut, D. Sunar Cerci, B. Tali, H. Topakli, M. Vergili, I. V. Akin, B. Bilin, S. Bilmis, H. Gamsizkan, G. Karapinar, K. Ocalan, S. Sekmen, U. E. Surat, M. Yalvac, M. Zeyrek, E. Gülmez, B. Isildak, M. Kaya, O. Kaya, K. Cankocak, F. I. Vardarlı, L. Levchuk, P. Sorokin, J. J. Brooke, E. Clement, D. Cussans, H. Flacher, R. Frazier, J. Goldstein, M. Grimes, G. P. Heath, H. F. Heath, J. Jacob, L. Kreczko, C. Lucas, Z. Meng, D. M. Newbold, S. Paramesvaran, A. Poll, S. Senkin, V. J. Smith, T. Williams, K. W. Bell, A. Belyaev, C. Brew, R. M. Brown, D. J. A. Cockerill, J. A. Coughlan, K. Harder, S. Harper, E. Olaiya, D. Petyt, C. H. Shepherd-Themistocleous, A. Thea, I. R. Tomalin, W. J. Womersley, S. D. Worm, M. Baber, R. Bainbridge, O. Buchmuller, D. Burton, D. Colling, N. Cripps, M. Cutajar, P. Dauncey, G. Davies, M. Della Negra, P. Dunne, W. Ferguson, J. Fulcher, D. Futyan, A. Gilbert, G. Hall, G. Iles, M. Jarvis, G. Karapostoli, M. Kenzie, R. Lane, R. Lucas, L. Lyons, A.-M. Magnan, S. Malik, B. Mathias, J. Nash, A. Nikitenko, J. Pela, M. Pesaresi, K. Petridis, D. M. Raymond, S. Rogerson, A. Rose, C. Seez, P. Sharp, A. Tapper, M. Vazquez Acosta, T. Virdee, S. C Zenz, J. E. Cole, P. R. Hobson, A. Khan, P. Kyberd, D. Leggat, D. Leslie, W. Martin, I. D. Reid, P. Symonds, L. Teodorescu, M. Turner, J. Dittmann, K. Hatakeyama, A. Kasmi, H. Liu, T. Scarborough, O. Charaf, S. I. Cooper, C. Henderson, P. Rumerio, A. Avetisyan, T. Bose, C. Fantasia, P. Lawson, C. Richardson, J. Rohlf, D. Sperka, J. St. John, L. Sulak, J. Alimena, E. Berry, S. Bhattacharya, G. Christopher, D. Cutts, Z. Demiragli, N Dhingra, A. Ferapontov, A. Garabedian, U. Heintz, G. Kukartsev, E. Laird, G. Landsberg, M. Luk, M. Narain, M. Segala, T. Sinthuprasith, T. Speer, J. Swanson, R. Breedon, G. Breto, M. Calderon De La Barca Sanchez, S. Chauhan, M. Chertok, J. Conway, R. Conway, P. T. Cox, R. Erbacher, M. Gardner, W. Ko, R. Lander, T. Miceli, M. Mulhearn, D. Pellett, J. Pilot, F. Ricci-Tam, M. Searle, S. Shalhout, J. Smith, M. Squires, D. Stolp, M. Tripathi, S. Wilbur, R. Yohay, R. Cousins, P. Everaerts, C. Farrell, J. Hauser, M. Ignatenko, G. Rakness, E. Takasugi, V. Valuev, M. Weber, J. Babb, K. Burt, R. Clare, J. Ellison, J. W. Gary, G. Hanson, J. Heilman, M. Ivova Rikova, P. Jandir, E. Kennedy, F. Lacroix, H. Liu, O. R. Long, A. Luthra, M. Malberti, H. Nguyen, M. Olmedo Negrete, A. Shrinivas, S. Sumowidagdo, S. Wimpenny, W. Andrews, J. G. Branson, G. B. Cerati, S. Cittolin, R. T. D’Agnolo, D. Evans, A. Holzner, R. Kelley, D. Klein, M. Lebourgeois, J. Letts, I. Macneill, D. Olivito, S. Padhi, C. Palmer, M. Pieri, M. Sani, V. Sharma, S. Simon, E. Sudano, M. Tadel, Y. Tu, A. Vartak, C. Welke, F. Würthwein, A. Yagil, J. Yoo, D. Barge, J. Bradmiller-Feld, C. Campagnari, T. Danielson, A. Dishaw, K. Flowers, M. Franco Sevilla, P. Geffert, C. George, F. Golf, L. Gouskos, J. Incandela, C. Justus, N. Mccoll, J. Richman, D. Stuart, W. To, C. West, A. Apresyan, A. Bornheim, J. Bunn, Y. Chen, E. Di Marco, J. Duarte, A. Mott, H. B. Newman, C. Pena, C. Rogan, M. Spiropulu, V. Timciuc, R. Wilkinson, S. Xie, R. Y. Zhu, V. Azzolini, A. Calamba, B. Carlson, T. Ferguson, Y. Iiyama, M. Paulini, J. Russ, H. Vogel, I. Vorobiev, J. P. Cumalat, W. T. Ford, A. Gaz, E. Luiggi Lopez, U. Nauenberg, J. G. Smith, K. Stenson, K. A. Ulmer, S. R. Wagner, J. Alexander, A. Chatterjee, J. Chu, S. Dittmer, N. Eggert, N. Mirman, G. Nicolas Kaufman, J. R. Patterson, A. Ryd, E. Salvati, L. Skinnari, W. Sun, W. D. Teo, J. Thom, J. Thompson, J. Tucker, Y. Weng, L. Winstrom, P. Wittich, D. Winn, S. Abdullin, M. Albrow, J. Anderson, G. Apollinari, L. A. T. Bauerdick, A. Beretvas, J. Berryhill, P. C. Bhat, K. Burkett, J. N. Butler, H. W. K. Cheung, F. Chlebana, S. Cihangir, V. D. Elvira, I. Fisk, J. Freeman, Y. Gao, E. Gottschalk, L. Gray, D. Green, S. Grünendahl, O. Gutsche, J. Hanlon, D. Hare, R. M. Harris, J. Hirschauer, B. Hooberman, S. Jindariani, M. Johnson, U. Joshi, K. Kaadze, B. Klima, B. Kreis, S. Kwan, J. Linacre, D. Lincoln, R. Lipton, T. Liu, J. Lykken, K. Maeshima, J. M. Marraffino, V. I. Martinez Outschoorn, S. Maruyama, D. Mason, P. McBride, K. Mishra, S. Mrenna, Y. Musienko, S. Nahn, C. Newman-Holmes, V. O’Dell, O. Prokofyev, E. Sexton-Kennedy, S. Sharma, A. Soha, W. J. Spalding, L. Spiegel, L. Taylor, S. Tkaczyk, N. V. Tran, L. Uplegger, E. W. Vaandering, R. Vidal, A. Whitbeck, J. Whitmore, F. Yang, D. Acosta, P. Avery, D. Bourilkov, M. Carver, T. Cheng, D. Curry, S. Das, M. De Gruttola, G. P. Di Giovanni, R. D. Field, M. Fisher, I. K. Furic, J. Hugon, J. Konigsberg, A. Korytov, T. Kypreos, J. F. Low, K. Matchev, P. Milenovic, G. Mitselmakher, L. Muniz, A. Rinkevicius, L. Shchutska, M. Snowball, J. Yelton, M. Zakaria, S. Hewamanage, S. Linn, P. Markowitz, G. Martinez, J. L. Rodriguez, T. Adams, A. Askew, J. Bochenek, B. Diamond, J. Haas, S. Hagopian, V. Hagopian, K. F. Johnson, H. Prosper, V. Veeraraghavan, M. Weinberg, M. M. Baarmand, M. Hohlmann, H. Kalakhety, F. Yumiceva, M. R. Adams, L. Apanasevich, V. E. Bazterra, D. Berry, R. R. Betts, I. Bucinskaite, R. Cavanaugh, O. Evdokimov, L. Gauthier, C. E. Gerber, D. J. Hofman, S. Khalatyan, P. Kurt, D. H. Moon, C. O’Brien, C. Silkworth, P. Turner, N. Varelas, E. A. Albayrak, B. Bilki, W. Clarida, K. Dilsiz, F. Duru, M. Haytmyradov, J.-P. Merlo, H. Mermerkaya, A. Mestvirishvili, A. Moeller, J. Nachtman, H. Ogul, Y. Onel, F. Ozok, A. Penzo, R. Rahmat, S. Sen, P. Tan, E. Tiras, J. Wetzel, T. Yetkin, K. Yi, B. A. Barnett, B. Blumenfeld, S. Bolognesi, D. Fehling, A. V. Gritsan, P. Maksimovic, C. Martin, M. Swartz, P. Baringer, A. Bean, G. Benelli, C. Bruner, R. P. Kenny, M. Malek, M. Murray, D. Noonan, S. Sanders, J. Sekaric, R. Stringer, Q. Wang, J. S. Wood, A. F. Barfuss, I. Chakaberia, A. Ivanov, S. Khalil, M. Makouski, Y. Maravin, L. K. Saini, S. Shrestha, N. Skhirtladze, I. Svintradze, J. Gronberg, D. Lange, F. Rebassoo, D. Wright, A. Baden, A. Belloni, B. Calvert, S. C. Eno, J. A. Gomez, N. J. Hadley, R. G. Kellogg, T. Kolberg, Y. Lu, M. Marionneau, A. C. Mignerey, K. Pedro, A. Skuja, M. B. Tonjes, S. C. Tonwar, A. Apyan, R. Barbieri, G. Bauer, W. Busza, I. A. Cali, M. Chan, L. Di Matteo, V. Dutta, G. Gomez Ceballos, M. Goncharov, D. Gulhan, M. Klute, Y. S. Lai, Y.-J. Lee, A. Levin, P. D. Luckey, T. Ma, C. Paus, D. Ralph, C. Roland, G. Roland, G. S. F. Stephans, F. Stöckli, K. Sumorok, D. Velicanu, J. Veverka, B. Wyslouch, M. Yang, A. S. Yoon, M. Zanetti, V. Zhukova, B. Dahmes, A. De Benedetti, A. Gude, S. C. Kao, K. Klapoetke, Y. Kubota, J. Mans, N. Pastika, R. Rusack, A. Singovsky, N. Tambe, J. Turkewitz, J. G. Acosta, L. M. Cremaldi, R. Kroeger, S. Oliveros, L. Perera, D. A. Sanders, D. Summers, E. Avdeeva, K. Bloom, S. Bose, D. R. Claes, A. Dominguez, R. Gonzalez Suarez, J. Keller, D. Knowlton, I. Kravchenko, J. Lazo-Flores, S. Malik, F. Meier, G. R. Snow, J. Dolen, A. Godshalk, I. Iashvili, S. Jain, A. Kharchilava, A. Kumar, S. Rappoccio, G. Alverson, E. Barberis, D. Baumgartel, M. Chasco, J. Haley, A. Massironi, D. Nash, T. Orimoto, D. Trocino, R -J. Wang, D. Wood, J. Zhang, A. Anastassov, K. A. Hahn, A. Kubik, L. Lusito, N. Mucia, N. Odell, B. Pollack, A. Pozdnyakov, M. Schmitt, S. Stoynev, K. Sung, M. Velasco, S. Won, A. Brinkerhoff, K. M. Chan, A. Drozdetskiy, M. Hildreth, C. Jessop, D. J. Karmgard, N. Kellams, K. Lannon, W. Luo, S. Lynch, N. Marinelli, T. Pearson, M. Planer, R. Ruchti, N. Valls, M. Wayne, M. Wolf, A. Woodard, L. Antonelli, J. Brinson, B. Bylsma, L. S. Durkin, S. Flowers, C. Hill, R. Hughes, K. Kotov, T. Y. Ling, D. Puigh, M. Rodenburg, G. Smith, B. L. Winer, H. Wolfe, H. W. Wulsin, O. Driga, P. Elmer, P. Hebda, A. Hunt, S. A. Koay, P. Lujan, D. Marlow, T. Medvedeva, M. Mooney, J. Olsen, P. Piroué, X. Quan, H. Saka, D. Stickland, C. Tully, J. S. Werner, A. Zuranski, E. Brownson, H. Mendez, J. E. Ramirez Vargas, V. E. Barnes, D. Benedetti, G. Bolla, D. Bortoletto, M. De Mattia, Z. Hu, M. K. Jha, M. Jones, K. Jung, M. Kress, N. Leonardo, D. Lopes Pegna, V. Maroussov, P. Merkel, D. H. Miller, N. Neumeister, B. C. Radburn-Smith, X. Shi, I. Shipsey, D. Silvers, A. Svyatkovskiy, F. Wang, W. Xie, L. Xu, H. D. Yoo, J. Zablocki, Y. Zheng, N. Parashar, J. Stupak, A. Adair, B. Akgun, K. M. Ecklund, F. J. M. Geurts, W. Li, B. Michlin, B. P. Padley, R. Redjimi, J. Roberts, J. Zabel, B. Betchart, A. Bodek, R. Covarelli, P. de Barbaro, R. Demina, Y. Eshaq, T. Ferbel, A. Garcia-Bellido, P. Goldenzweig, J. Han, A. Harel, A. Khukhunaishvili, G. Petrillo, D. Vishnevskiy, R. Ciesielski, L. Demortier, K. Goulianos, G. Lungu, C. Mesropian, S. Arora, A. Barker, J. P. Chou, C. Contreras-Campana, E. Contreras-Campana, D. Duggan, D. Ferencek, Y. Gershtein, R. Gray, E. Halkiadakis, D. Hidas, S. Kaplan, A. Lath, S. Panwalkar, M. Park, R. Patel, S. Salur, S. Schnetzer, S. Somalwar, R. Stone, S. Thomas, P. Thomassen, M. Walker, K. Rose, S. Spanier, A. York, O. Bouhali, A. Castaneda Hernandez, R. Eusebi, W. Flanagan, J. Gilmore, T. Kamon, V. Khotilovich, V. Krutelyov, R. Montalvo, I. Osipenkov, Y. Pakhotin, A. Perloff, J. Roe, A. Rose, A. Safonov, T. Sakuma, I. Suarez, A. Tatarinov, N. Akchurin, C. Cowden, J. Damgov, C. Dragoiu, P. R. Dudero, J. Faulkner, K. Kovitanggoon, S. Kunori, S. W. Lee, T. Libeiro, I. Volobouev, E. Appelt, A. G. Delannoy, S. Greene, A. Gurrola, W. Johns, C. Maguire, Y. Mao, A. Melo, M. Sharma, P. Sheldon, B. Snook, S. Tuo, J. Velkovska, M. W. Arenton, S. Boutle, B. Cox, B. Francis, J. Goodell, R. Hirosky, A. Ledovskoy, H. Li, C. Lin, C. Neu, J. Wood, C. Clarke, R. Harr, P. E. Karchin, C. Kottachchi Kankanamge Don, P. Lamichhane, J. Sturdy, D. A. Belknap, D. Carlsmith, M. Cepeda, S. Dasu, L. Dodd, S. Duric, E. Friis, R. Hall-Wilton, M. Herndon, A. Hervé, P. Klabbers, A. Lanaro, C. Lazaridis, A. Levine, R. Loveless, A. Mohapatra, I. Ojalvo, T. Perry, G. A. Pierro, G. Polese, I. Ross, T. Sarangi, A. Savin, W. H. Smith, C. Vuosalo, N. Woods, The CMS Collaboration

**Affiliations:** 1 Yerevan Physics Institute, Yerevan, Armenia; 2 Institut für Hochenergiephysik der OeAW, Wien, Austria; 3 National Centre for Particle and High Energy Physics, Minsk, Belarus; 4 Universiteit Antwerpen, Antwerpen, Belgium; 5 Vrije Universiteit Brussel, Brussel, Belgium; 6 Université Libre de Bruxelles, Bruxelles, Belgium; 7 Ghent University, Ghent, Belgium; 8 Université Catholique de Louvain, Louvain-la-Neuve, Belgium; 9 Université de Mons, Mons, Belgium; 10 Centro Brasileiro de Pesquisas Fisicas, Rio de Janeiro, Brazil; 11 Universidade do Estado do Rio de Janeiro, Rio de Janeiro, Brazil; 12 Universidade Estadual Paulista, Universidade Federal do ABC, São Paulo, Brazil; 13 Institute for Nuclear Research and Nuclear Energy, Sofia, Bulgaria; 14 University of Sofia, Sofia, Bulgaria; 15 Institute of High Energy Physics, Beijing, China; 16 State Key Laboratory of Nuclear Physics and Technology, Peking University, Beijing, China; 17 Universidad de Los Andes, Bogota, Colombia; 18 University of Split, Faculty of Electrical Engineering, Mechanical Engineering and Naval Architecture, Split, Croatia; 19 University of Split, Faculty of Science, Split, Croatia; 20 Institute Rudjer Boskovic, Zagreb, Croatia; 21 University of Cyprus, Nicosia, Cyprus; 22 Charles University, Prague, Czech Republic; 23 Academy of Scientific Research and Technology of the Arab Republic of Egypt, Egyptian Network of High Energy Physics, Cairo, Egypt; 24 National Institute of Chemical Physics and Biophysics, Tallinn, Estonia; 25 Department of Physics, University of Helsinki, Helsinki, Finland; 26 Helsinki Institute of Physics, Helsinki, Finland; 27 Lappeenranta University of Technology, Lappeenranta, Finland; 28 DSM/IRFU, CEA/Saclay, Gif-sur-Yvette, France; 29 Laboratoire Leprince-Ringuet, Ecole Polytechnique, IN2P3-CNRS, Palaiseau, France; 30 Institut Pluridisciplinaire Hubert Curien, Université de Strasbourg, Université de Haute Alsace Mulhouse, CNRS/IN2P3, Strasbourg, France; 31 Centre de Calcul de l’Institut National de Physique Nucleaire et de Physique des Particules, CNRS/IN2P3, Villeurbanne, France; 32 Institut de Physique Nucléaire de Lyon, Université de Lyon, Université Claude Bernard Lyon 1, CNRS-IN2P3, Villeurbanne, France; 33 Institute of High Energy Physics and Informatization, Tbilisi State University, Tbilisi, Georgia; 34 RWTH Aachen University, I. Physikalisches Institut, Aachen, Germany; 35 RWTH Aachen University, III. Physikalisches Institut A, Aachen, Germany; 36 RWTH Aachen University, III. Physikalisches Institut B, Aachen, Germany; 37 Deutsches Elektronen-Synchrotron, Hamburg, Germany; 38 University of Hamburg, Hamburg, Germany; 39 Institut für Experimentelle Kernphysik, Karlsruhe, Germany; 40 Institute of Nuclear and Particle Physics (INPP), NCSR Demokritos, Aghia Paraskevi, Greece; 41 University of Athens, Athens, Greece; 42 University of Ioánnina, Ioánnina, Greece; 43 Wigner Research Centre for Physics, Budapest, Hungary; 44 Institute of Nuclear Research ATOMKI, Debrecen, Hungary; 45 University of Debrecen, Debrecen, Hungary; 46 National Institute of Science Education and Research, Bhubaneswar, India; 47 Panjab University, Chandigarh, India; 48 University of Delhi, Delhi, India; 49 Saha Institute of Nuclear Physics, Kolkata, India; 50 Bhabha Atomic Research Centre, Mumbai, India; 51 Tata Institute of Fundamental Research, Mumbai, India; 52 Institute for Research in Fundamental Sciences (IPM), Tehran, Iran; 53 University College Dublin, Dublin, Ireland; 54 INFN Sezione di Bari, Università di Bari, Politecnico di Bari, Bari, Italy; 55 INFN Sezione di Bologna, Università di Bologna, Bologna, Italy; 56 INFN Sezione di Catania, Università di Catania, CSFNSM, Catania, Italy; 57 INFN Sezione di Firenze, Università di Firenze, Firenze, Italy; 58 INFN Laboratori Nazionali di Frascati, Frascati, Italy; 59 INFN Sezione di Genova, Università di Genova, Genoa, Italy; 60 INFN Sezione di Milano-Bicocca, Università di Milano-Bicocca, Milan, Italy; 61 INFN Sezione di Napoli, Università di Napoli ’Federico II’, Università della Basilicata (Potenza), Università G. Marconi (Roma), Naples, Italy; 62 INFN Sezione di Padova, Università di Padova, Università di Trento (Trento), Padua, Italy; 63 INFN Sezione di Pavia, Università di Pavia, Pavia, Italy; 64 INFN Sezione di Perugia, Università di Perugia, Perugia, Italy; 65 INFN Sezione di Pisa, Università di Pisa, Scuola Normale Superiore di Pisa, Pisa, Italy; 66 INFN Sezione di Roma, Università di Roma, Rome, Italy; 67 INFN Sezione di Torino, Università di Torino, Università del Piemonte Orientale (Novara), Torino, Italy; 68 INFN Sezione di Trieste, Università di Trieste, Trieste, Italy; 69 Kangwon National University, Chunchon, Korea; 70 Kyungpook National University, Daegu, Korea; 71 Chonbuk National University, Jeonju, Korea; 72 Chonnam National University, Institute for Universe and Elementary Particles, Kwangju, Korea; 73 Korea University, Seoul, Korea; 74 University of Seoul, Seoul, Korea; 75 Sungkyunkwan University, Suwon, Korea; 76 Vilnius University, Vilnius, Lithuania; 77 National Centre for Particle Physics, Universiti Malaya, Kuala Lumpur, Malaysia; 78 Centro de Investigacion y de Estudios Avanzados del IPN, Mexico City, Mexico; 79 Universidad Iberoamericana, Mexico City, Mexico; 80 Benemerita Universidad Autonoma de Puebla, Puebla, Mexico; 81 Universidad Autónoma de San Luis Potosí, San Luis Potosí, Mexico; 82 University of Auckland, Auckland, New Zealand; 83 University of Canterbury, Christchurch, New Zealand; 84 National Centre for Physics, Quaid-I-Azam University, Islamabad, Pakistan; 85 National Centre for Nuclear Research, Swierk, Poland; 86 Institute of Experimental Physics, Faculty of Physics, University of Warsaw, Warsaw, Poland; 87 Laboratório de Instrumentação e Física Experimental de Partículas, Lisbon, Portugal; 88 Joint Institute for Nuclear Research, Dubna, Russia; 89 Petersburg Nuclear Physics Institute, Gatchina (St. Petersburg), Russia; 90 Institute for Nuclear Research, Moscow, Russia; 91 Institute for Theoretical and Experimental Physics, Moscow, Russia; 92 P. N. Lebedev Physical Institute, Moscow, Russia; 93 Skobeltsyn Institute of Nuclear Physics, Lomonosov Moscow State University, Moscow, Russia; 94 State Research Center of Russian Federation, Institute for High Energy Physics, Protvino, Russia; 95 University of Belgrade, Faculty of Physics and Vinca Institute of Nuclear Sciences, Belgrade, Serbia; 96 Centro de Investigaciones Energéticas Medioambientales y Tecnológicas (CIEMAT), Madrid, Spain; 97 Universidad Autónoma de Madrid, Madrid, Spain; 98 Universidad de Oviedo, Oviedo, Spain; 99 Instituto de Física de Cantabria (IFCA), CSIC-Universidad de Cantabria, Santander, Spain; 100 CERN, European Organization for Nuclear Research, Geneva, Switzerland; 101 Paul Scherrer Institut, Villigen, Switzerland; 102 Institute for Particle Physics, ETH Zurich, Zurich, Switzerland; 103 Universität Zürich, Zurich, Switzerland; 104 National Central University, Chung-Li, Taiwan; 105 National Taiwan University (NTU), Taipei, Taiwan; 106 Chulalongkorn University, Faculty of Science, Department of Physics, Bangkok, Thailand; 107 Cukurova University, Adana, Turkey; 108 Physics Department, Middle East Technical University, Ankara, Turkey; 109 Bogazici University, Istanbul, Turkey; 110 Istanbul Technical University, Istanbul, Turkey; 111 National Scientific Center, Kharkov Institute of Physics and Technology, Kharkov, Ukraine; 112 University of Bristol, Bristol, UK; 113 Rutherford Appleton Laboratory, Didcot, UK; 114 Imperial College, London, UK; 115 Brunel University, Uxbridge, UK; 116 Baylor University, Waco, USA; 117 The University of Alabama, Tuscaloosa, USA; 118 Boston University, Boston, USA; 119 Brown University, Providence, USA; 120 University of California, Davis, USA; 121 University of California, Los Angeles, USA; 122 University of California, Riverside, Riverside, USA; 123 University of California, San Diego, La Jolla, USA; 124 University of California, Santa Barbara, Santa Barbara, USA; 125 California Institute of Technology, Pasadena, USA; 126 Carnegie Mellon University, Pittsburgh, USA; 127 University of Colorado at Boulder, Boulder, USA; 128 Cornell University, Ithaca, USA; 129 Fairfield University, Fairfield, USA; 130 Fermi National Accelerator Laboratory, Batavia, USA; 131 University of Florida, Gainesville, USA; 132 Florida International University, Miami, USA; 133 Florida State University, Tallahassee, USA; 134 Florida Institute of Technology, Melbourne, USA; 135 University of Illinois at Chicago (UIC), Chicago, USA; 136 The University of Iowa, Iowa City, USA; 137 Johns Hopkins University, Baltimore, USA; 138 The University of Kansas, Lawrence, USA; 139 Kansas State University, Manhattan, USA; 140 Lawrence Livermore National Laboratory, Livermore, USA; 141 University of Maryland, College Park, USA; 142 Massachusetts Institute of Technology, Cambridge, USA; 143 University of Minnesota, Minneapolis, USA; 144 University of Mississippi, Oxford, USA; 145 University of Nebraska-Lincoln, Lincoln, USA; 146 State University of New York at Buffalo, Buffalo, USA; 147 Northeastern University, Boston, USA; 148 Northwestern University, Evanston, USA; 149 University of Notre Dame, Notre Dame, USA; 150 The Ohio State University, Columbus, USA; 151 Princeton University, Princeton, USA; 152 University of Puerto Rico, Mayaguez, USA; 153 Purdue University, West Lafayette, USA; 154 Purdue University Calumet, Hammond, USA; 155 Rice University, Houston, USA; 156 University of Rochester, Rochester, USA; 157 The Rockefeller University, New York, USA; 158 Rutgers, The State University of New Jersey, Piscataway, USA; 159 University of Tennessee, Knoxville, USA; 160 Texas A&M University, College Station, USA; 161 Texas Tech University, Lubbock, USA; 162 Vanderbilt University, Nashville, USA; 163 University of Virginia, Charlottesville, USA; 164 Wayne State University, Detroit, USA; 165 University of Wisconsin, Madison, USA; 166 CERN, Geneva, Switzerland

## Abstract

The purely electroweak (EW) cross section for the production of two jets in association with a Z boson, in proton–proton collisions at $\sqrt{s}=8\,\text {TeV}$, is measured using data recorded by the CMS experiment at the CERN LHC, corresponding to an integrated luminosity of 19.7$\,\text {fb}^\text {-1}$. The electroweak cross section for the $\ell \ell \mathrm {jj}$ final state (with $\ell = \mathrm {e}$ or $\mu$ and j representing the quarks produced in the hard interaction) in the kinematic region defined by $M_{\ell \ell } >50$ $\,\text {GeV}$, $M_\mathrm {jj} >120$ $\,\text {GeV}$, transverse momentum $p_\mathrm {T j}> 25$ $\,\text {GeV}$, and pseudorapidity $|\eta _\mathrm {j} |< 5$, is found to be $\sigma _\mathrm {EW}(\ell \ell \mathrm {jj})=174 \pm 15\,\text {(stat)}\pm 40\,\text {(syst)}\text {\,fb}$, in agreement with the standard model prediction. The associated jet activity of the selected events is studied, in particular in a signal-enriched region of phase space, and the measurements are found to be in agreement with QCD predictions.

## Introduction

The production of a ${\mathrm{Z}}$ boson in association with two jets in proton–proton (pp) collisions is dominated by a mixture of electroweak (EW) and strong processes of order $\alpha _\mathrm {EW}^2\alpha _\mathrm {S}^2$. For ${\mathrm{Z}}\rightarrow \ell \ell$ leptonic decays, such events are referred to as “Drell–Yan (DY) + jets” or $\mathrm {DY}\,{\mathrm{Z}}\mathrm {jj}$ events.

**Fig. 1:**
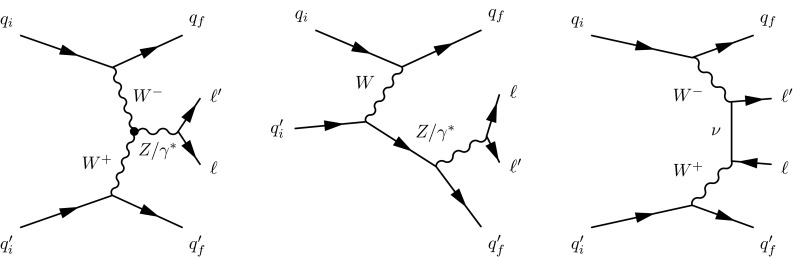
Representative Feynman diagrams for dilepton production in association with two jets from purely electroweak contributions: (*left*) vector boson fusion, (*middle*) bremsstrahlung-like, and (*right*) multiperipheral production

Purely electroweak $\ell \ell \mathrm {jj}$ production contributing to the same final state is expected at order $\alpha _\mathrm {EW}^4$, resulting in a comparatively small cross section [1]. This process is however predicted to have a distinctive signature of two jets of very high energy and large jj invariant mass, $M_\mathrm {jj}$, separated by a large rapidity interval that can be occupied by the two charged leptons and where extra gluon emission is suppressed [2, 3]. We refer to jets produced through the fragmentation of the outgoing quarks in pure EW processes as “tagging jets”, and to the process from which they originate as “$\mathrm {EW}\,{\mathrm{Z}}\mathrm {jj}$ ”. Figure 1 shows representative Feynman diagrams for the $\mathrm {EW}\,{\mathrm{Z}}\mathrm {jj}$ processes, namely (left) vector boson fusion (VBF), (middle) bremsstrahlung-like, and (right) multiperipheral production. Detailed calculations reveal the presence of a large negative interference between the pure VBF process and the two other categories [1, 3]. These diagrams represent the signal (S) in the data.

**Fig. 2:**
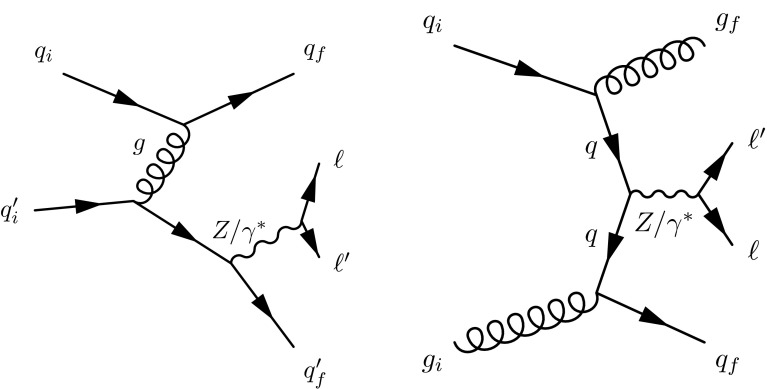
Representative diagrams for order $\alpha _\mathrm {S}^2$ corrections to DY production that comprise the main background (B) in this study

For inclusive $\ell \ell \mathrm {jj}$ final states, some of the diagrams with same initial- and final-state particles and quantum numbers can interfere, even if they do not involve exclusively EW interactions. Figure 2 (left) shows one example of order $\alpha _\mathrm {S}^2$ corrections to DY production that have the same initial and final state as those in Fig. 1. A different order $\alpha _\mathrm {S}^2$ correction that does not interfere with the EW signal, is shown in Fig. 2 (right).

The study of $\mathrm {EW}\,{\mathrm{Z}}\mathrm {jj}$ processes is part of a more general investigation of standard model (SM) vector boson fusion and scattering processes that include the Higgs boson [4–6] and searches for physics beyond the standard model [7, 8]. When isolated from the backgrounds, the properties of $\mathrm {EW}\,{\mathrm{Z}}\mathrm {jj}$ events can be compared with SM predictions. Probing the jet activity in the selected events in particular can shed light on the selection (or vetoing) of additional parton radiation to the tagging jets [9, 10].

At the CERN LHC, the $\mathrm {EW}\,{\mathrm{Z}}\mathrm {jj}$ process was first measured by the CMS experiment using pp collisions at $\sqrt{s}=7\,\text {TeV}$ [11], and more recently by the ATLAS experiment at $\sqrt{s}=8\,\text {TeV}$ [12]. Both results have been found to agree with the expectations of the SM. Our present work reflects the measurement at CMS using pp collision data collected at $\sqrt{s}=8$ $\,\text {TeV}$during 2012 that correspond to an integrated luminosity of 19.7$\,\text {fb}^\text {-1}$. As the signal-to-background ratio for the measurement is small, different methods are used to enhance the signal fraction, to confirm the presence of the signal, and to measure the cross section. Besides the two multivariate analyses, based on the methods developed for the 7$\,\text {TeV}$ analysis [11], a new method is presented, using a model of the main background based on real pp collisions. The analysis of the 8$\,\text {TeV}$ data, offers the opportunity of reducing the uncertainties of the 7$\,\text {TeV}$ measurements, given the larger integrated luminosity, and to add robustness to the results with the new data-based method.

This paper is organised as follows: Sect. 2 describes the experimental apparatus and Sect. 3 the simulations. Event selection procedures are described in Sect. 4, and Sect. 5 discusses the selection efficiencies and background models in control regions. Section 6 details the strategies adopted in our analysis to extract the signal from the data, and the corresponding systematic uncertainties are summarised in Sect. 7. The results obtained are presented in Sect. 8, and we conclude with a study of jet properties in a $\mathrm {DY}\,{\mathrm{Z}}\mathrm {jj}$-dominated control region, as well as in a high-purity, $\mathrm {EW}\,{\mathrm{Z}}\mathrm {jj}$-enriched region in Sect. 9. Finally, a brief summary of the results is given in Sect. 10.

## The CMS detector

The central feature of the CMS apparatus is a superconducting solenoid of 6$\text {\,m}$ internal diameter, providing a magnetic field of 3.8$\text {\,T}$. The solenoid volume contains a silicon pixel and strip tracker, a lead tungstate crystal electromagnetic calorimeter (ECAL), and a brass/scintillator hadron calorimeter (HCAL), each composed of a barrel and two endcap sections. Muons are measured in gas-ionisation tracking detectors embedded in the steel flux-return yoke outside the solenoid. Extensive forward calorimetry complements the coverage provided by the barrel and endcap detectors.

The silicon tracker consists of 1440 silicon pixel modules and 15 148 silicon strip detector modules, located in the field of the superconducting solenoid. It measures charged particles within $|\eta |< 2.5$, providing an impact parameter resolution of ${\approx }15\upmu$ and a transverse momentum ($p_{\mathrm {T}}$) resolution of about 1.5 % for $p_{\mathrm {T}} =100\,\text {GeV}$ particles.

The energy of electrons is measured after combining the information from the ECAL and the tracker, whereas their direction is measured by the tracker. The invariant mass resolution for ${\mathrm{Z}}\rightarrow \mathrm {e}\mathrm {e}$ decays is 1.6 % when both electrons are in the ECAL barrel, and 2.6 % when both electrons are in the ECAL endcap [13]. Matching muons to tracks measured in the silicon tracker yields a $p_{\mathrm {T}}$ resolution between $1$ and 10 %, for $p_{\mathrm {T}}$ values up to 1$\,\text {TeV}$. The jet energy resolution (JER) is typically ${\approx }15\,\%$ at 50$\,\text {GeV}$, 8 % at 100$\,\text {GeV}$, and 4 % at 1$\,\text {TeV}$ [14].

## Simulation of signal and background events

Signal events are simulated at leading order (LO) using the MadGraph (v5.1.3.30) Monte Carlo (MC) generator [15, 16], interfaced to pythia (v6.4.26) [17] for parton showering (PS) and hadronisation. The CTEQ6L1 [18] parton distribution functions (PDF) are used to generate the event, the factorisation ($\mu _F$) and renormalisation ($\mu _R$) scales being both fixed to be equal to the ${\mathrm{Z}}$-boson mass [19]. The underlying event is modelled with the so-called $Z2^{*}$ tune [20]. The simulation does not include the generation of extra partons at matrix-element level. In the kinematic region defined by dilepton mass $M_{\ell \ell } >50\,\text {GeV}$, parton transverse momentum $p_\mathrm {T j}> 25\,\text {GeV}$, parton pseudorapidity $\vert \eta _\mathrm{j}\vert < 5$, diparton mass $M_\mathrm{jj} > 120\,\text {GeV}$, and angular separation $\Delta R_\mathrm{jj}=\sqrt{{(\Delta \eta _\mathrm{jj})^2+(\Delta \phi _\mathrm{jj})^2}}>0.5$, where $\Delta \eta _\mathrm {jj}$ and $\Delta \phi _\mathrm {jj}$ are the differences in pseudorapidity and azimuthal angle between the tagging partons, the cross section in the $\ell \ell$jj final state (with $\ell$ = e or $\mu$) is expected to be $\sigma _\mathrm {LO}(\mathrm {EW}~\ell \ell \mathrm {jj})=208^{+8}_{-9}\,\text {(scale)}\pm 7\,\text {(PDF)}\text {\,fb}$, where the first uncertainty is obtained by changing simultaneously $\mu _F$ and $\mu _R$ by factors of $2$ and $1/2$, and the second from the uncertainties in the PDFs which has been estimated following the pdf4lhc prescription [18, 21–24]. The LO signal cross section and kinematic distributions estimated with MadGraph are found to be in good agreement with the LO predictions of the vbfnlo generator (v.2.6.3) [25–27].

Background DY events are also generated with MadGraph using a LO matrix element (ME) calculation that includes up to four partons generated from quantum chromodynamics (QCD) interactions. The ME-PS matching is performed following the ktMLM prescription [28, 29]. The dilepton DY production for $M_{\ell \ell }>50\,\text {GeV}$ is normalised to $\sigma _\text {th}(\mathrm {DY})=3.504\text {\,nb}$, as computed at next-to-next-leading order (NNLO) with fewz [30].

The evaluation of the interference between $\mathrm {EW}\,{\mathrm{Z}}\mathrm {jj}$ and $\mathrm {DY}\,{\mathrm{Z}}\mathrm {jj}$ processes, relies on the predictions obtained with MadGraph. Three samples, one of pure signal, one pure background, and one including both $\alpha _\mathrm {EW}^4$ and $\alpha _\mathrm {EW}^2\alpha _\mathrm {S}^2$ contributions are generated for this purpose. The differential cross sections are compared and used to estimate the expected interference contributions at the parton level.

Other residual background is expected from events with two leptons of same flavour with accompanying jets in the final state. Production of ${\mathrm{t}}\overline{{\mathrm{t}}}$ events is generated with MadGraph, including up to three extra partons, and normalised to the NNLO with next-to-next-to-leading-logarithmic corrections to an inclusive cross section of 245.8 $\text {\,pb}$ [31]. Single-top-quark processes are modelled at next-to-leading order (NLO) with powheg  [32–36] and normalised, respectively, to cross sections of $22\pm 2$, $86\pm 3$, and $5.6\pm 0.2\,\text {\,pb}$ for the tW, $t$-, and $s$- channel production [37, 38]. Diboson production processes $\mathrm {W}\mathrm {W}$, $\mathrm {W}{\mathrm{Z}}$, and ${\mathrm{Z}}{\mathrm{Z}}$ are generated with MadGraph and normalised, respectively, to the cross sections of 59.8, 33.2, and 17.7 $\text {\,pb}$, computed at NNLO [39] and with mcfm  [40]. Throughout this paper we use the abbreviation VV when referring to the sum of the processes which yield two vector bosons.

The production of a $\mathrm {W}$ boson in association with jets, where the $\mathrm {W}$ decays to a charged lepton and a neutrino, is generated with MadGraph, and normalised to a total cross section of 36.3 nb, computed at NNLO with Fewz. Multijet QCD processes are also studied in simulation, but are found to yield negligible contributions to the selected events.

A detector simulation based on Geant4 (v.9.4p03) [41, 42] is applied to all the generated signal and background samples. The presence of multiple pp interactions in the same beam crossing (pileup) is incorporated by simulating additional interactions (both in-time and out-of-time with the collision) with a multiplicity that matches the one observed in data. The average number of pileup events is estimated as $\approx$21 interactions per bunch crossing.

## Reconstruction and selection of events

The event selection is optimised to identify dilepton final states with two isolated, high-$p_{\mathrm {T}}$ leptons, and at least two high-$p_{\mathrm {T}}$ jets. Dilepton triggers are used to acquire the data, where one lepton is required to have $p_{\mathrm {T}} >17 \,\text {GeV}$ and the other to have $p_{\mathrm {T}} >8 \,\text {GeV}$. Electron-based triggers include additional isolation requirements, both in the tracker detectors and in the calorimeters. A single-isolated-muon trigger, with a requirement of $p_{\mathrm {T}} >24 \,\text {GeV}$, is used to complement the dimuon trigger and increase the efficiency of the selection.

Electrons are reconstructed from clusters of energy depositions in the ECAL that match tracks extrapolated from the silicon tracker [43]. Muons are reconstructed by fitting trajectories based on hits in the silicon tracker and in the outer muon system [44]. Reconstructed electron or muon candidates are required to have $p_{\mathrm {T}} >20\,\text {GeV}$. Electron candidates are required to be reconstructed within $|\eta |\le 2.5$, excluding the CMS barrel-to-endcap transition region of the ECAL [45], and muon candidates are required to be reconstructed in the fiducial region $|\eta |\le 2.4$ of the tracker system. The track associated to a lepton candidate is required to have both its transverse and longitudinal impact parameters compatible with the position of the primary vertex (PV) of the event. The PV for each event is defined as the one with the largest $\sum p_{\mathrm {T}} ^2$, where the sum runs over all the tracks used to fit the vertex. A particle-based relative isolation parameter is computed for each lepton, and corrected on an event-by-event basis for contributions from pileup. The particle candidates used to compute the isolation variable are reconstructed with the particle flow algorithm which will be detailed later. We require that the sum of the scalar $p_{\mathrm {T}}$ of all particle candidates reconstructed in an isolation cone with radius $R=\sqrt{{(\Delta \eta )^{2}+(\Delta \phi )^{2}}}<0.4$ around the lepton’s momentum vector is $<$10 or $<$12 % of the electron or muon $p_{\mathrm {T}}$ value, respectively. The two leptons with opposite electric charge and with highest $p_{\mathrm {T}}$ are chosen to form the dilepton pair. Same-flavour dileptons (ee or $\mu \mu$) compatible with ${\mathrm{Z}}\rightarrow \ell \ell$ decays are then selected by requiring $|M_{\mathrm{Z}}-M_{\ell \ell }|<15 \,\text {GeV}$, where $M_{\mathrm{Z}}$ is the mass of the ${\mathrm{Z}}$ boson [19].

Two types of jets are used in the analysis: “jet-plus-track” (JPT) [46] and particle-flow (PF) [14] jets. Both cases use the anti-$k_{\mathrm {T}}$ algorithm [47, 48] with a distance parameter of 0.5 to define jets. The information from the ECAL, HCAL and tracker are used by both algorithms in distinct ways. The JPT algorithm improves the energy response and resolution of calorimeter jets by incorporating additional tracking information. For JPT jets the associated tracks are classified as in-cone or out-of-cone if they point to within or outside the jet cone around the jet axis at the surface of the calorimeter. The momenta of both in-cone and out-of-cone tracks are then added to the energy of the associated calorimeter jet and for in-cone tracks the expected average energy deposition in the calorimeters is subtracted based on the momentum of the track. The direction of the jet axis is also corrected by the algorithm. As a result, the JPT algorithm improves both the energy and the direction of the jet. The PF algorithm [49, 50] combines the information from all relevant CMS sub-detectors to identify and reconstruct particle candidates in the event: muons, electrons, photons, charged hadrons, and neutral hadrons. The PF jets are constructed by clustering these particle candidates and the jet momentum is defined as the vectorial sum of the momenta of all particle candidates. An area-based correction is applied to both JPT and PF jets, to account for the extra energy that is clustered through in-time pileup [51, 52]. Jet energy scale (JES) and resolution (JER) for JPT and PF jets are derived from simulation and confirmed with in situ measurements of the $p_{\mathrm {T}}$ balance observed in exclusive dijet and ${\mathrm{Z}}$/photon+jet events. The simulation is corrected so that it describes the JER from real data. Additional selection criteria are applied to each event to remove spurious jet-like features originating from isolated noise patterns in certain HCAL regions. Jet identification criteria are furthermore applied to remove contributions from jets clustered from pileup events. These criteria are described in more detail in Ref. [53]. As will be detailed in Sect. 5.1, the efficiency of these algorithms has been measured in data and it is observed to be compatible with the expectations from simulation across the full pseudorapidity range used in the analysis.

In the preselection of events we require at least two jets with $p_{\mathrm {T}} >30\,\text {GeV}$ and ${|\eta |\le 4.7}$. The two jets of highest $p_{\mathrm {T}}$ jets are defined as the tagging jets. For the measurement of the cross section, we require the leading jet to have $p_{\mathrm {T}} >50\,\text {GeV}$ and the dijet invariant mass $M_\mathrm {jj}>200\,\text {GeV}$. Other selection requirements will be described below, as they depend on the analysis.

## Control regions for jets and modelling of background

In our analysis, we select control regions for different purposes: to validate the calibrated jet energy response and efficiencies of jet-identification criteria, to estimate the backgrounds and to verify the agreement between data and estimates of background. The following details the result of these cross-checks.

**Fig. 3:**
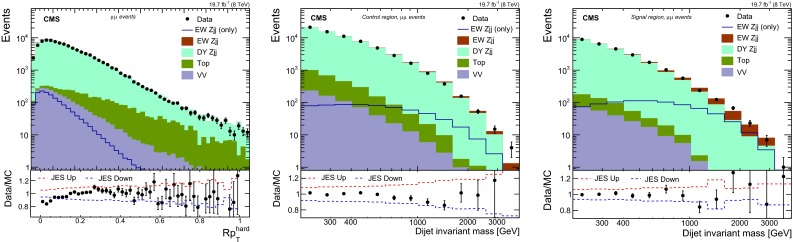
Distribution for (*left*) $Rp_{\mathrm {T}} ^\text {hard}$ and $M_\mathrm {jj}$ for $\mu \mu$ events with (*middle*) $Rp_{\mathrm {T}} ^\text {hard}\ge 0.14$ (control region) and (*right*) $Rp_{\mathrm {T}} ^\text {hard}<0.14$ (signal region). The contributions from the different background sources and the signal are shown stacked, with data points superimposed. The *panels* below the distributions show the ratio between the data and expectations as well as the uncertainty envelope for the impact of the uncertainty of the JES

**Fig. 4:**
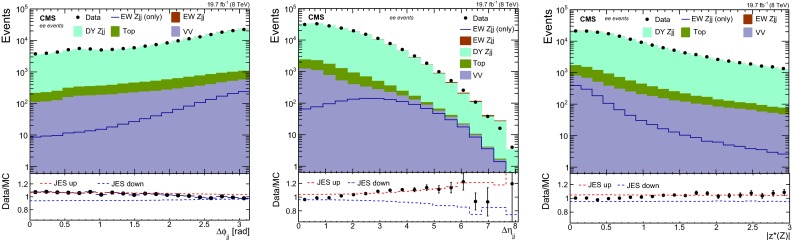
Distribution for (*left*) the difference in the azimuthal angle and (*middle*) difference in the pseudorapidity of the tagging jets for ee events, with $Rp_{\mathrm {T}} ^\text {hard}\ge 0.14$. The $z^*$ distribution (*right*) is shown for the same category of events. The *panels* below the distributions show the ratio between the data and expectations as well as the uncertainty envelope for the impact of the uncertainty of the JES

### Jet identification and response

Events with either a ${\mathrm{Z}}\rightarrow \mu \mu$ or a photon candidate, produced in association with a single jet with $p_{\mathrm {T}}$ $>30\,\text {GeV}$, are used as one of the control samples in this analysis. The ${\mathrm{Z}}$ candidate or the photon, and the associated jet are required to have $|\Delta \phi (\text {jet},{\mathrm{Z}}\text { or }\gamma ) |>2.7\text {\,rad}$. These events enable a measure of the efficiency of the algorithms used to reject calorimeter noise and pileup-induced jets, and to check the jet energy response.

**Fig. 5:**
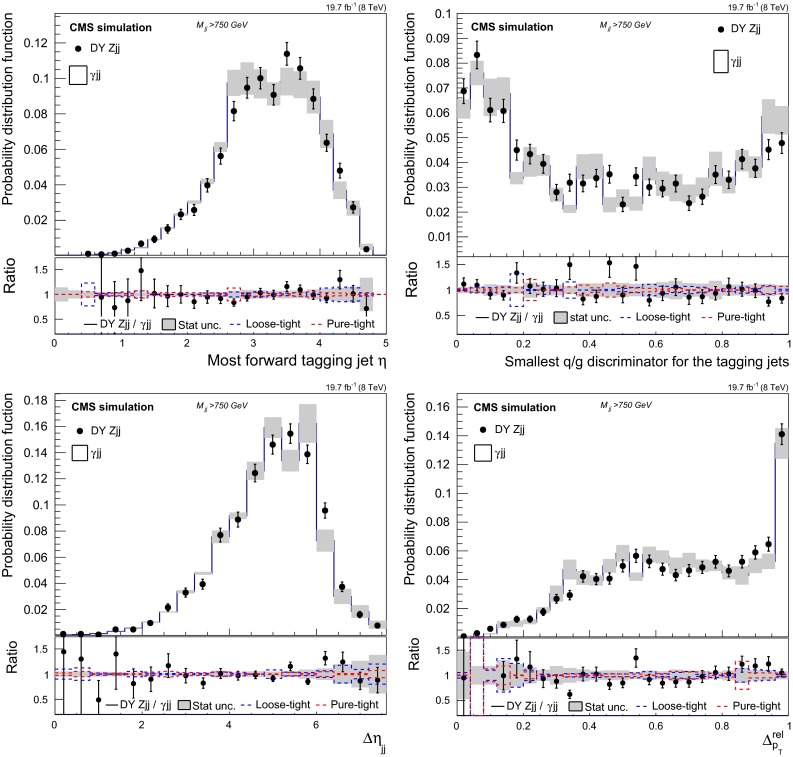
Comparison of the $\mathrm {DY}\,{\mathrm{Z}}\mathrm {jj}$ distributions with the prediction from the photon control sample, for simulated events with $M_\mathrm {jj}>750\,\text {GeV}$. The *upper left* subfigure shows the distributions in the pseudorapidity $\eta$ of the most forward tagging jet and the *upper right* shows the smallest q/g discriminant of the two tagging jets. The *lower left* shows the pseudorapidity separation $\Delta \eta _\mathrm {jj}$ and the *lower right* the relative $p_{\mathrm {T}}$ balance of the tagging jets $\Delta ^\text {rel}_{p_{\mathrm {T}}}$. The DY $\gamma \mathrm {jj}$ distribution contains the contribution from prompt and misidentified photons as estimated from simulation and it is compared to the simulated $\mathrm {DY}\,{\mathrm{Z}}\mathrm {jj}$ sample in the *top panel* of each subfigure. The *bottom panels* show the ratio between the $\mathrm {DY}\,{\mathrm{Z}}\mathrm {jj}$ distribution and the photon-based prediction, and includes the different sources of estimated total uncertainty in the background shape from the photon control sample. (See text for specification of impact of loose, tight and pure photons)

**Fig. 6:**
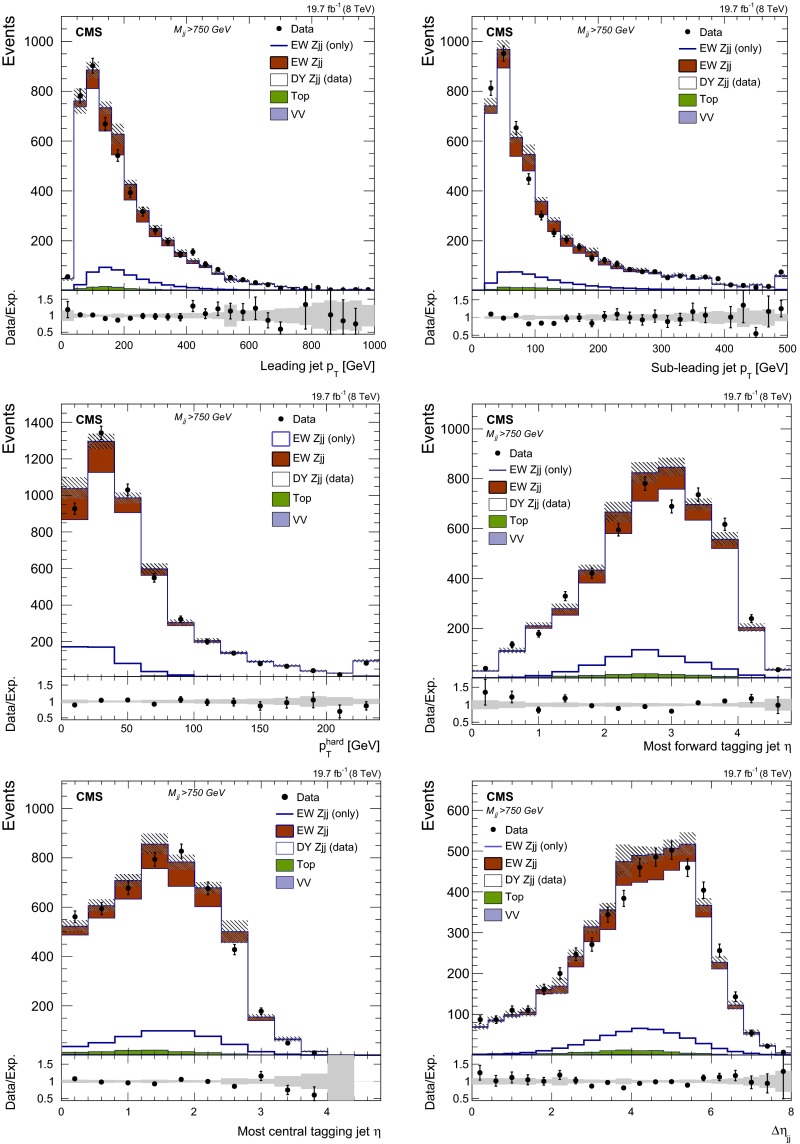
Distributions for the tagging jets for $M_\mathrm {jj}>750\,\text {GeV}$ in the combined dielectron and dimuon event sample: (*upper left*) $p_{\mathrm {T}}$ of the leading jet, (*upper right*) $p_{\mathrm {T}}$ of the sub-leading jet, (*middle left*) hard process $p_{\mathrm {T}}$ (dijet+${\mathrm{Z}}$ system), (*middle right*) $\eta$ of the most forward jet, (*lower left*) $\eta$ of the most central jet and (*lower right*) $\Delta \eta _\mathrm {jj}$ of the tagging jets. In the *top panels*, the contributions from the different background sources and the signal are shown stacked being data superimposed. In all plots the signal shape is also superimposed separately as a thick line. The *bottom panels* show the ratio between data and total prediction. The total uncertainty assigned to the $\mathrm {DY}\,{\mathrm{Z}}\mathrm {jj}$ background estimate from $\gamma \mathrm {jj}$ control sample in data is shown in all panels as a *shaded grey band*

The jet identification criteria are based on the fractions of the jet energy deposited in different calorimeter elements [14]. Besides calorimetric noise, pileup events result in additional reconstructed jets. Such pileup jets can be rejected through a multivariate analysis based on the kinematics of the jet, on the topological configuration of its constituents, and on the fraction of tracks in the jet, associated to other reconstructed PVs in the same event [53]. The efficiency of both jet identification and pileup rejection is measured in the control sample, and determined to be >$98\,\%$ for both JPT and PF jets. The dependence of this efficiency on $\eta$ agrees with that predicted in MC simulation. The residual $\eta$-dependent difference is used to assign a systematic uncertainty in the selected signal.

The same control sample is also used to verify the jet energy response [14], which is defined from the ratio $\left[ p_{\mathrm {T}} (\text {jet})/p_{\mathrm {T}} ({\mathrm{Z}}\text { or }\gamma )\right]$. The double ratio of the response in data and in simulation, i.e. $\big [p_{\mathrm {T}} (\text {jet})/p_{\mathrm {T}} ({\mathrm{Z}}\text { or }\gamma )\big ]_\text {data}/ \big [p_{\mathrm {T}} (\text {jet})/p_{\mathrm {T}} ({\mathrm{Z}}\text { or }\gamma )\big ]_\mathrm {MC}$, provides a residual uncertainty that is assigned as a systematic source of uncertainty to the measurement. Although partially covered by the JES uncertainties, this procedure considers possible residual uncertainties in the particular phase-space regions selected in our analysis. This evaluation is crucial for the most forward region of $\eta$, where the uncertainties in response are large. The double ratio defined above is observed to be close to unity except for a small loss in response ($\approx$5 %) observed in the region where the tracker has no acceptance and where there is a transition from the endcap to the forward hadron calorimeters of CMS ($2.7<|\eta |<3.2$).

### Discriminating gluons from quarks

Jets in signal events are expected to originate from quarks while for background events it is more probable that jets are initiated by a gluon emitted from a radiative QCD process. A quark–gluon (q/g) discriminant [11] is evaluated for the two tagging jets with the intent of distinguishing the nature of each jet.

The q/g discriminant exploits differences in the showering and fragmentation of gluons and quarks, making use of the internal jet-composition and structure observables. The jet particle multiplicity and the maximum energy fraction carried by a particle inside the jet are used. In addition the q/g discriminant makes use of the following variables, computed using the weighted $p_{\mathrm {T}} ^2$-sum of the particles inside a jet: the jet constituents’ major root-mean-square (RMS) distance in the $\eta$–$\phi$ plane, the jet constituents’ minor RMS distance in the $\eta$–$\phi$ plane, and the jet asymmetry pull. Further details can be found in [54, 55].

The variables are used as an input to a likelihood-ratio discriminant that is trained using the tmva package [56] on gluon and quark jets from simulated dijet events. To improve the separation power, all variables are corrected for their pileup contamination using the same estimator for the average energy density from pileup interactions [51, 52], as previously defined in Sect. 4. The performance of the q/g discriminant has been evaluated and validated using independent, exclusive samples of ${\mathrm{Z}}$+jet and dijet data [54]. The use of the gluon–quark likelihood discriminator leads to a decrease of the statistical uncertainty of the measured signal by about 5 %.

### Modeling background

Alternative background models are explored for the dominant $\mathrm {DY}\,{\mathrm{Z}}\mathrm {jj}$ background. Given that the majority of the $\ell \ell \mathrm {jj}$ final states are produced through $\mathrm {DY}\,{\mathrm{Z}}\mathrm {jj}$ processes it is crucial to have different handles on the behavior of this process, in particular, in the signal phase space region.

**Simulation-based prediction for background**

The effect of virtual corrections to the MadGraph-based (Born-level) description of $\mathrm {DY}\,{\mathrm{Z}}\mathrm {jj}$ is studied using mcfm. Comparisons are made between the predictions of mcfm parton-level distributions with NLO and LO calculations and these studies provide a dynamic NLO to LO scale factor (K-factor) as a function of $M_\mathrm {jj}$ and of the difference between the rapidity of the ${\mathrm{Z}}$ boson and the average rapidity of the two tagging jets, i.e.

1 $$\begin{aligned} y^*=y_{{\mathrm{Z}}}-\frac{1}{2}(y_{\mathrm {j}_1}+y_{\mathrm {j}_2}). \end{aligned}$$

The K-factor is observed to have a minor dependence on $M_\mathrm {jj}$, but to increase steeply with $|y^* |$, and a correction greater than 10 %, relative to the signal, is obtained for $|y^* |>1.2$. As a consequence, an event selection of $|y^* |<1.2$ is introduced in the $\mathrm {DY}\,{\mathrm{Z}}\mathrm {jj}$ simulation-based analyses. Finally, the difference between the nominal MadGraph prediction and the one obtained after reweighting it with the dynamic K-factor, on an event-by-event basis, is assigned as a systematic uncertainty for the $\mathrm {DY}\,{\mathrm{Z}}\mathrm {jj}$ background prediction from simulation.

For the selection of the signal-region in the analysis where $\mathrm {DY}\,{\mathrm{Z}}\mathrm {jj}$ is based on simulation we make use of an event balance variable, $Rp_{\mathrm {T}} ^\text {hard}$, defined as

2 $$\begin{aligned} Rp_{\mathrm {T}} ^\text {hard}= \frac{| \mathbf {p}_{\mathrm {T} \mathrm {j}_1}+\mathbf {p}_{\mathrm {T} \mathrm {j}_2}+\mathbf {p}_{\mathrm {T} {\mathrm{Z}}} |}{ |\mathbf {p}_{\mathrm {T} \mathrm {j}_1} | +|\mathbf {p}_{\mathrm {T} \mathrm {j}_2} | + |\mathbf {p}_{\mathrm {T} {\mathrm{Z}}} | } \!=\! \frac{ |\mathbf {p}_{\mathrm {T}}^\text {hard} |}{ |\mathbf {p}_{\mathrm {T} \mathrm {j}_1} | +|\mathbf {p}_{\mathrm {T} \mathrm {j}_2} | + |\mathbf {p}_{\mathrm {T} {\mathrm{Z}}} | },\nonumber \\ \end{aligned}$$

where the numerator is the estimator of the $p_{\mathrm {T}}$ for the hard process, i.e. $p_{\mathrm {T}} ^\text {hard}$. The distribution of the $Rp_{\mathrm {T}} ^\text {hard}$ variable is shown in Fig. 3 (left), where data and simulation are found to be in agreement with each other. It can be seen, from the same figure, that the variable is robust against the variation of JES according to its uncertainty. We apply a requirement of $Rp_{\mathrm {T}} ^\text {hard}<0.14$ to select the signal region and the events failing this requirement are used as a control region for the analyses. The cut is motivated by the fact that the signal is expected to have the ${\mathrm{Z}}$ boson balanced with respect to the dijet system in the transverse plane. The events which fail this requirement are used as control region for the modelling of the background. The $M_\mathrm {jj}$ distribution in dimuon events for the signal and control regions is shown in Fig. 3, (middle) and (right), correspondingly. The reweighting of the $\mathrm {DY}\,{\mathrm{Z}}\mathrm {jj}$ background is applied to the simulation, as described above. Data and predictions are found to be in agreement with each other.

Figure 4 shows distributions for angle-related variables. Fair agreement is observed for the absolute differences in the azimuthal angle ($\Delta \phi _\mathrm {jj}$) and in the pseudorapidity ($\Delta \eta _\mathrm {jj}$) of the tagging jets which are shown on the left and middle, respectively. The $z^*$ variable [10] is shown in Fig. 4 (right), and it is defined as

3 $$\begin{aligned} z^*=\frac{ y^* }{ \Delta y_\mathrm {jj} }. \end{aligned}$$

Data is verified to be in good agreement with the prediction for the distribution in $$ z^*$$ variable.

**Table 1:** Comparison of the selections and variables used in three different analyses. The variables marked with the black circle are used in the discriminant of the indicated analysis

Analysis	*A*	*B*	*C*
Channels	ee, $\mu \mu$	$\mu \mu$	ee, $\mu \mu$
			binned in $M_\mathrm {jj}$
Selection	$p_{\mathrm {T} \mathrm {j}_1,\mathrm {j}_2}>50,30\,\text {GeV}$		
	$Rp_{\mathrm {T}} ^\text {hard}<0.14$		$p_{\mathrm {T}{\mathrm{Z}}}>50\,\text {GeV}$
	$|y^{*} |<1.2$		$|y_{\mathrm{Z}} |<1.4442$
	$M_\mathrm {jj}>200\,\text {GeV}$		$M_\mathrm {jj}>450\,\text {GeV}$
Jets	PF	JPT	PF
Variables used			
$M_\mathrm {jj}$	$\bullet$	$\bullet$	$\bullet$
$p_{\mathrm {T} \mathrm {j}_1},p_{\mathrm {T} \mathrm {j}_2}$		$\bullet$	$\bullet$
$\eta _{\mathrm {j}_1},\eta _{\mathrm {j}_2}$			$\bullet$
$\Delta _\text {rel}(\mathrm {jj})=\frac{\vert \mathbf {p}_{\mathrm {T} \mathrm {j}_1}+\mathbf {p}_{\mathrm {T} \mathrm {j}_2}\vert }{p_{\mathrm {T} \mathrm {j}_1}+p_{\mathrm {T} \mathrm {j}_2}}$			$\bullet$
$\Delta \eta _\mathrm {jj}$		$\bullet$	
$\vert \eta _{\mathrm {j}_1}\vert +\vert \eta _{\mathrm {j}_2}\vert$	$\bullet$	$\bullet$	$\bullet$
$\Delta \phi _\mathrm {jj}$		$\bullet$	$\bullet$
$\Delta \phi _{{\mathrm{Z}},{\mathrm {j}_1}}$		$\bullet$	
$y_{\mathrm{Z}}$	$\bullet$	$\bullet$	
$z^*_{\mathrm{Z}}$	$\bullet$		
$p_{\mathrm {T} {\mathrm{Z}}}$	$\bullet$	$\bullet$	
$Rp_{\mathrm {T}} ^\text {hard}$		$\bullet$	
q/g discriminator	$\bullet$		$\bullet$
$\mathrm {DY}\,{\mathrm{Z}}\mathrm {jj}$ model	MC-based	MC-based	From data

**Data-based prediction for background**

The diagrams contributing to the production of a photon and two jets ($\gamma \mathrm {jj}$) are expected to resemble those involved in the production of $\mathrm {DY}\,{\mathrm{Z}}\mathrm {jj}$ (see Fig. 2). Thus, we build a data-based model for the shapes of the distributions of the kinematic observables of the tagging jets from $\gamma \mathrm {jj}$ events selected in a similar way as the ${\mathrm{Z}}\mathrm {jj}$ ones. The differences, specific to the ${\mathrm{Z}}$ or photon-sample, are expected to be mitigated by reweighting the $p_{\mathrm {T}}$ of the photons to the $p_{\mathrm {T}}$ of the ${\mathrm{Z}}$ candidates. From simulation, we expect that the differences between the $\gamma$ and ${\mathrm{Z}}$ masses do not contribute significantly when matching the dijet kinematics between the two samples after $M_\mathrm {jj}>2M_{{\mathrm{Z}}}$ is required. Given that the photon sample is affected by multijet production, and that the selection of the low-$p_{\mathrm {T}}$ region in data is also affected by very large prescaling at the trigger stages, we impose tighter kinematic constraints on the reconstructed boson, with respect to the ones applied at pre-selection (Sect. 4). To match effectively the ${\mathrm{Z}}$ and photon kinematics, we require $p_{\mathrm {T}} ({\mathrm{Z}}\text { or }\gamma )>50\,\text {GeV}$ and rapidity $\vert y({\mathrm{Z}}\text { or }\gamma )\vert <1.44$. The rapidity requirement corresponds to the physical boundary of the central (barrel) region of the CMS ECAL [45].

**Fig. 7:**
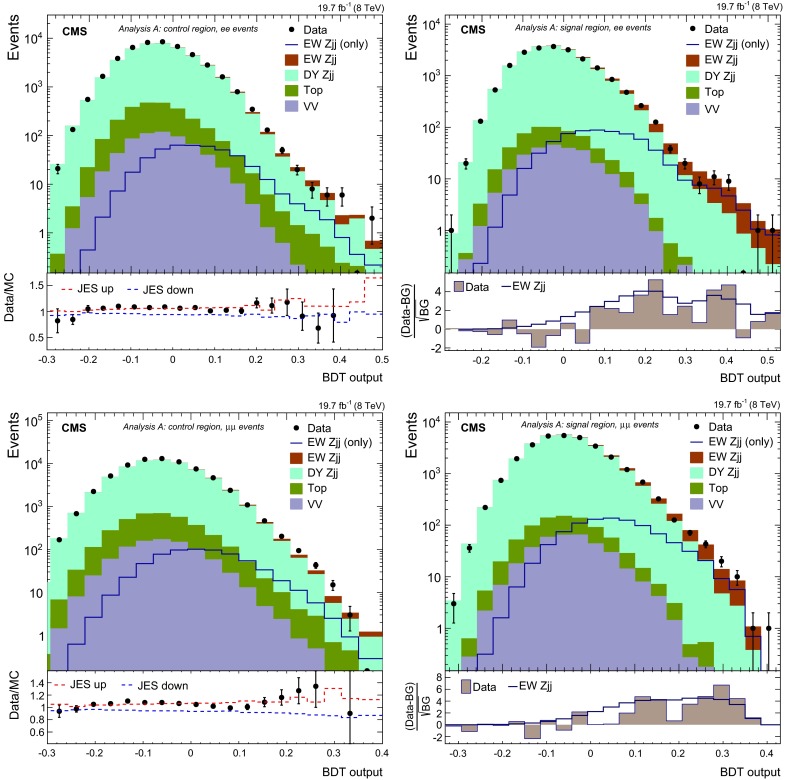
Distributions for the BDT discriminants in ee (*top row*) and $\mu \mu$ (*bottom row*) events, used by analysis A. The distributions obtained in the control regions are shown at the *left* while the ones obtained in the signal region are shown at the *right*. The ratios for data to MC simulations are given in the *bottom panels* in the *left column*, showing the impact of changes in JES by $\pm$1 SD. The *bottom panels* of the *right column* show the differences between data or the expected $\mathrm {EW}\,{\mathrm{Z}}\mathrm {jj}$ contribution with respect to the background (BG)

The method is checked in simulation by characterising the $\mathrm {DY}\,{\mathrm{Z}}\mathrm {jj}$ or direct photon events in different physical regions defined according to the reconstructed $M_\mathrm {jj}$ and comparing both distributions. Figure 5 illustrates the compatibility of simulated events with a high dijet invariant mass. Good agreement is found for the $\eta$ of the most forward jet, the $\Delta \eta _\mathrm {jj}$ variable and the ratio between the $p_{\mathrm {T}}$ of the dijet system to the scalar sum of the tagging jets’ $p_{\mathrm {T}}$,

4 $$\begin{aligned} \Delta ^\text {rel}_{p_{\mathrm {T}}}= \frac{|\mathbf {p}_{\mathrm {T} \mathrm {j}_1}+\mathbf {p}_{\mathrm {T} \mathrm {j}_2} |}{|\mathbf {p}_{\mathrm {T} \mathrm {j}_1} |+|\mathbf {p}_{\mathrm {T} \mathrm {j}_2} |}. \end{aligned}$$

The smallest of the quark/gluon discriminant value among the tagging jets is also found to be in agreement — Fig. 5 (top right). In general, the kinematics of the tagging jets predicted from the photon sample are found to be in agreement with those observed in DY ${\mathrm{Z}}$ events also for lower $M_\mathrm {jj}$ values. A similar conclusion holds for other global event observables inspected in the simulation, such as energy fluxes and angular correlations.

The result of the compatibility tests described above have the potential to yield a correction factor to be applied to the $\mathrm {DY}\,{\mathrm{Z}}\mathrm {jj}$ prediction from the photon data. However due to the limited statistics in our simulation and due to uncertainties in handling the simulation of residual background from multijet events in data, we have opted to use the simulation-based compatibility test results to assign, instead, an uncertainty in the final shape. We assign the difference in the compatibility tests relative to a pure prompt-photon possibility as one of the systematic uncertainties. The changes observed in the compatibility test, obtained after varying the PDF by its uncertainties synchronously in the two samples is also assigned as a source of uncertainty. In data, the difference between a “tight” and a “loose” photon selections is, furthermore, assigned as an extra source of systematic uncertainty. The selection is tightened by applying stricter requirements on the photon identification and isolation requirements. This prescription is adopted to cover possible effects from the contamination of multijet processes.

**Fig. 8:**
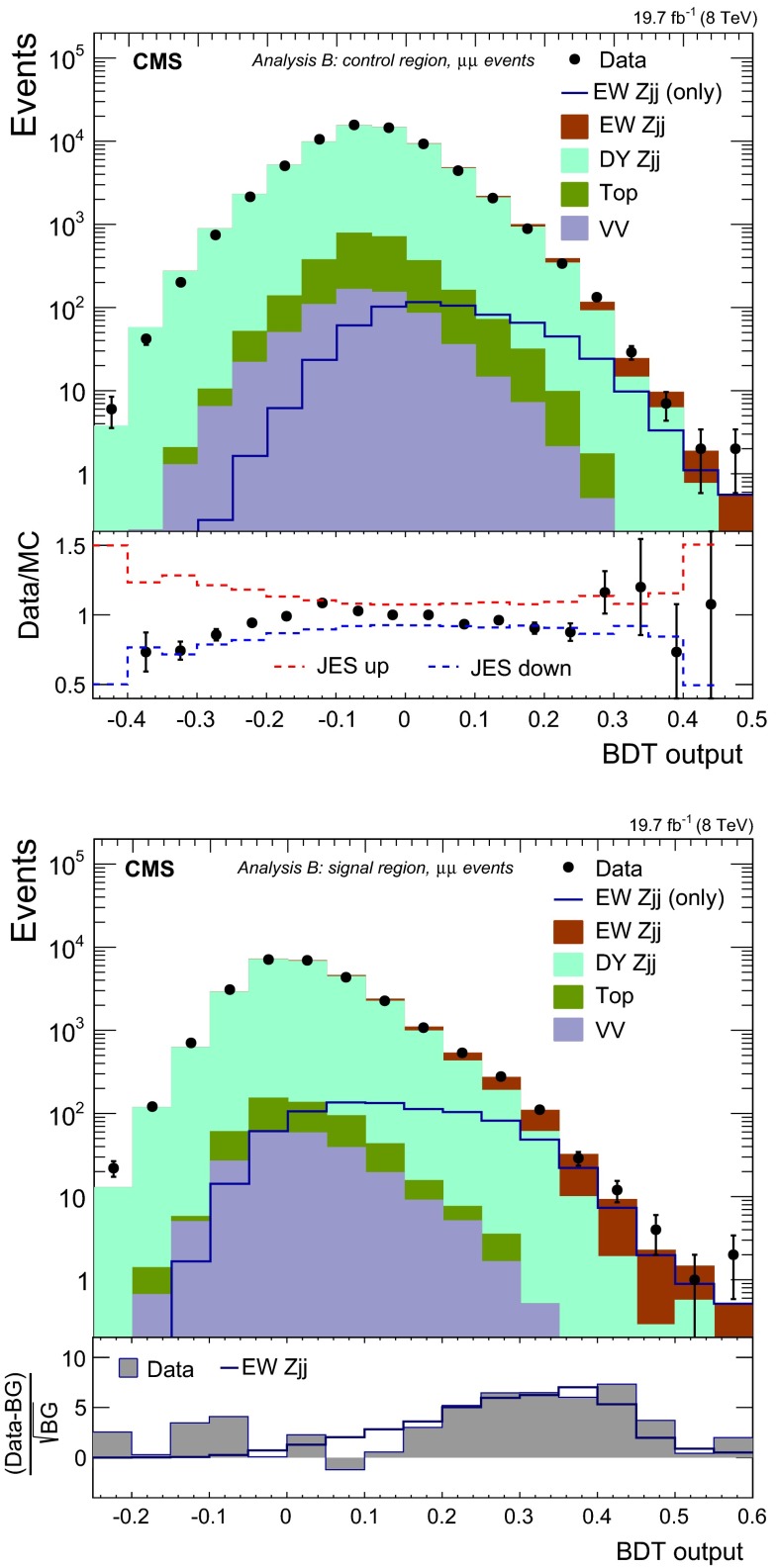
Distributions for the BDT discriminants in $\mu \mu$ events, for the control region (*top row*) and signal region (*bottom row*), used by analysis B. The ratio for data to MC simulations is given in the *bottom panel on the left*, showing the impact of changes in JES by $\pm$1 SD. The *bottom panel on the right* shows the difference between data or the expected $\mathrm {EW}\,{\mathrm{Z}}\mathrm {jj}$ contribution with respect to the background (BG)

The final distributions for $\mathrm {DY}\,{\mathrm{Z}}\mathrm {jj}$ events are obtained after subtracting a residual contamination from pure EW production of a photon in association with two jets ($\mathrm {EW}\,\gamma \mathrm {jj}$) [57]. The diagrams for the latter process are similar to the ones of Fig. 1 (left) and (middle), where the ${\mathrm{Z}}/\gamma ^*$ is now a real photon. For a fiducial phase space defined by $M_\mathrm {jj} >120\,\text {GeV}$, $p_\mathrm {T j}> 30\,\text {GeV}$, $|\eta _\mathrm {j} |< 5$, $p_{\mathrm {T} \gamma }>50\,\text {GeV}$ and $|\eta _\gamma |<1.5$, the production cross section of $\mathrm {EW}\,\gamma \mathrm {jj}$ process is expected to be 2.72 $\text {\,pb}$, based on the MadGraph generator. After event reconstruction and selection, we estimate the ratio of the number of $\mathrm {EW}\,\gamma \mathrm {jj}$ candidate events to the total number of photon events selected in data to be a factor of $\approx$5 times smaller than the ratio between the expected $\mathrm {EW}\,{\mathrm{Z}}\mathrm {jj}$ and $\mathrm {DY}\,{\mathrm{Z}}\mathrm {jj}$ yields. From simulations this ratio is expected to be independent of $M_\mathrm {jj}$. In the subtraction procedure, a 30 % normalisation uncertainty is assigned to this residual process, which corresponds to approximately twice the envelope of variations obtained for the cross section at NLO with vbfnlo, after tightening the selection criteria and changing the factorisation and renormalisation scales.

The results obtained when the data-based prediction, used to characterise the $\mathrm {DY}\,{\mathrm{Z}}\mathrm {jj}$ contribution to the reconstructed kinematics of the tagging jets in data, show a good agreement for different dijet invariant mass categories. Figure 6 illustrates the agreement observed for $M_\mathrm {jj}>750\,\text {GeV}$ in the distribution of different variables: (upper left) $p_{\mathrm {T}}$ of the leading jet, (upper right) $p_{\mathrm {T}}$ of the sub-leading jet, (middle left) hard process $p_{\mathrm {T}}$ (dijet+${\mathrm{Z}}$ system), (middle right) $\eta$ of the most forward jet, (lower left) $\eta$ of the most central jet and (lower right) $\Delta \eta _\mathrm {jj}$ of the tagging jets.

## Signal discriminants and extraction procedure

We use a multivariate analysis technique that provides separation of the $\mathrm {DY}\,{\mathrm{Z}}\mathrm {jj}$ and $\mathrm {EW}\,{\mathrm{Z}}\mathrm {jj}$ components of the inclusive $\ell \ell \mathrm {jj}$ spectrum. As discussed previously, the $\mathrm {EW}\,{\mathrm{Z}}\mathrm {jj}$ signal is characterised by a large $\Delta \eta _\mathrm {jj}$ jet separation that stems from the small-angle scattering of the two initial partons. Owing to both the topological configuration and the large $p_{\mathrm {T}}$ of the outgoing partons, the $M_\mathrm {jj}$ variable is also expected to be large. The evolution of $\Delta \eta _\mathrm {jj}$ with $M_\mathrm {jj}$ is expected to be different in signal and background events and therefore these characteristics are expected to yield the best separation power between the $\mathrm {EW}\,{\mathrm{Z}}\mathrm {jj}$ and the $\mathrm {DY}\,{\mathrm{Z}}\mathrm {jj}$ productions. In addition, one can exploit the fact that the ${\mathrm{Z}}$-boson candidate is expected to be produced centrally in the rapidity region defined by the two tagging jets and that the ${\mathrm{Z}}\mathrm {jj}$ system is approximately balanced in the transverse plane. As a consequence, we expect the signal to be found with lower values of both $y^*$ and $p_{\mathrm {T}} ^\text {hard}$, compared to the DY background. Other variables which can be used to enhance the separation are related to the kinematics of the event ($p_{\mathrm {T}}$, rapidity, and distance between the jets and/or the ${\mathrm{Z}}$ boson) or to the properties of the jets that are expected to be initiated by quarks. We combine these variables using three alternative multivariate analyses with the goal of cross-checking the final result. All three analyses make use of boosted decision tree (BDT) discriminators implemented using tmva package [56] to achieve the best expected separation between the $\mathrm {EW}\,{\mathrm{Z}}\mathrm {jj}$ and $\mathrm {DY}\,{\mathrm{Z}}\mathrm {jj}$ processes.

- Analysis A expands one of the procedures previously adopted for the 7$\,\text {TeV}$measurement [11]. It uses both dimuon and dielectron final states and PF jet reconstruction. A multivariate discriminator making use of the dijet and ${\mathrm{Z}}$ boson kinematics is built. A choice is made for variables which are robust against JES uncertainties. Extra discrimination information, related to the q/g nature of the jet, is included. All processes are modelled from simulation, and the description of each variable is verified by comparing data with the simulation-based expectations in control regions.
- Analysis B uses only the dimuon final state and the JPT jet reconstruction approach. It builds a discriminator which tries to profit from the full kinematics of the event including the tagging jets and the ${\mathrm{Z}}$ boson. Similarly to analysis A it expands one of the cross-check procedures previously adopted for the 7$\,\text {TeV}$measurement [11] and relies on simulation-based prediction of the backgrounds.
- Analysis C uses solely dijet-related variables in the multivariate discriminator and selects both the dimuon and dielectron final states with PF jets. Lepton-related selection variables are not used as the main background is derived from the photon control sample. In this analysis events are split in four categories for $M_\mathrm {jj}$ values in the intervals 450–550$\,\text {GeV}$, 550–750$\,\text {GeV}$, 750–1,000$\,\text {GeV}$, and above 1,000$\,\text {GeV}$, which have been chosen to have similar numbers of expected signal events.

Table 1 compares in more detail the three independent analyses A, B and C. From simulation, the statistical correlation between the analyses, if performed with the same final state, is estimated to be $\approx$60 %.

**Fig. 9:**
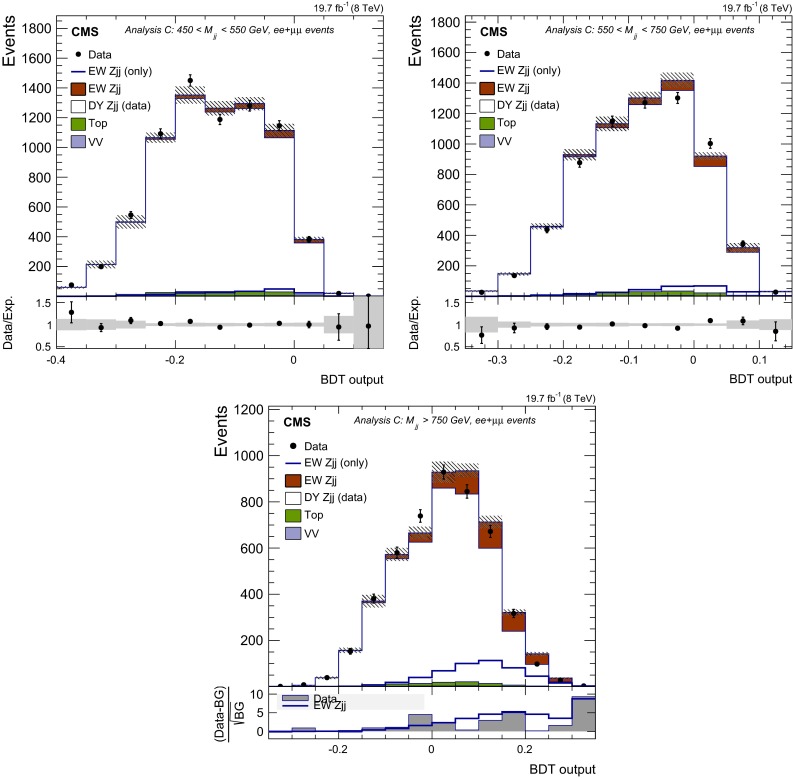
Distributions for the BDT discriminants in ee+$\mu \mu$ events for different $M_\mathrm {jj}$ categories, used in analysis C. The ratios at the bottom each subfigure of the top row gives the results of data to expectation for the two control regions of $M_\mathrm {jj}$. The lower panel of the bottom subfigure shows the difference between data or the expected $\mathrm {EW}\,{\mathrm{Z}}\mathrm {jj}$ contribution with respect to the background (BG)

Figures 7, 8 and 9 show the distributions of the discriminants for the three analyses. Good agreement is observed overall in both the signal and in the control regions which are defined according to the value of the $Rp_{\mathrm {T}} ^\text {hard}$ or $M_{\mathrm {jj}}$ variables (see Sect. 5.3).

Each analysis has a binned maximum likelihood formed from the expected rates for each process, as function of the value of the discriminant, which is used to fit simultaneously across the control and signal categories the strength modifiers for the $\mathrm {EW}\,{\mathrm{Z}}\mathrm {jj}$ and $\mathrm {DY}\,{\mathrm{Z}}\mathrm {jj}$ processes, $\mu = \sigma ({\mathrm {EW}~{\mathrm{Z}}\mathrm {jj}}) / \sigma _\mathrm {LO}({\mathrm {EW}~\ell \ell \mathrm {jj}})$ and $\upsilon = \sigma ({\mathrm {DY}})/\sigma _\text {th}({\mathrm {DY}})$. Nuisance parameters are added to modify the expected rates and shapes according to the estimate of the systematic uncertainties affecting the analysis and are mostly assumed to have a log-normal distribution.

The interference between the $\mathrm {EW}\,{\mathrm{Z}}\mathrm {jj}$ and the $\mathrm {DY}\,{\mathrm{Z}}\mathrm {jj}$ processes is taken into account in the fitting procedure. A parameterisation of the interference effects, as a function of the parton-level $M_\mathrm {jj}$ variable, is derived from the MadGraph simulation described in Sect. 3. The matrix elements for the $\mathrm {EW}\,{\mathrm{Z}}\mathrm {jj}$ and $\mathrm {DY}\,{\mathrm{Z}}\mathrm {jj}$ processes provide the total yields for the $\ell \ell \mathrm {jj}$ final state as

5 $$\begin{aligned} \hat{N}^{\ell \ell \mathrm {jj}}(\mu ,\upsilon )= \mu N_{\mathrm {EW}~{\mathrm{Z}}\mathrm {jj}} + \sqrt{\mu \upsilon } N_\mathrm {I} + \upsilon N_{\mathrm {DY}~{\mathrm{Z}}\mathrm {jj}}, \end{aligned}$$

where $N_{\mathrm {EW}~{\mathrm{Z}}\mathrm {jj}}$, $N_{\mathrm {DY}~{\mathrm{Z}}\mathrm {jj}}$ are the yields for the $\mathrm {EW}\,{\mathrm{Z}}\mathrm {jj}$ and $\mathrm {DY}\,{\mathrm{Z}}\mathrm {jj}$ processes, $N_\mathrm {I}$ is the expected contribution from the interference to the total yield, and $\mu$ and $\upsilon$ are the strength factors that modify the SM predictions. In the absence of signal (or background) the contribution from the interference term vanishes in Eq. (5).

The parameters of the model ($\mu$ and $\upsilon$) are determined maximising a likelihood ($\mathcal {L}$). Systematic uncertainties are incorporated in the fit by scanning the profile likelihood ratio $\lambda$, defined as

6 $$\begin{aligned} \lambda (\mu ,\nu ) = \frac{\mathcal {L}(\mu ,\nu ,\hat{\hat{\theta }})}{\mathcal {L}(\hat{\mu },\hat{\nu },\hat{\theta })}, \end{aligned}$$

where the denominator has estimators $\hat{\mu }$,$\hat{\nu }$ and $\hat{\theta }$ that maximise the likelihood, and the numerator has estimators $\hat{\hat{\theta }}$ that maximise the likelihood for the specified $\mu$ and $\nu$ strengths. The statistical methodology used is similar to the one used in the CMS Higgs analysis [5] using asymptotic formulas [58]. In this procedure some of the systematic uncertainties affecting the measurement of the signal strength are partially constrained. The $\mathrm {DY}\,{\mathrm{Z}}\mathrm {jj}$ strength is constrained by the uncertainties in analyses A and B and is free to change in C. In all cases the difference of the result relative to the one that would have been obtained without taking the interference term into account, is assigned as a systematic uncertainty of the measurement. This shall be discussed in more detail in the next section where the systematic uncertainties affecting our analysis are summarised.

**Table 2:** Summary of the relative variation of uncertainty sources (in %) considered for the evaluation of the systematic uncertainties in the different analyses. A filled or open circle signals whether that uncertainty affects the distribution or the absolute rate of a process in the fit, respectively. For some of the uncertainty sources “variable” is used to signal that the range is not unambiguously quantifiable by a range, as it depends on the value of the discriminants, event category and may also have a statistical component

Source	Shape	Methods A, B	Method C
Experimental			
Luminosity	$\circ$	2.6	
Trigger/selection	$\circ$	2–3	
JES and residual jet response	$\bullet$	1–10	
JER	$\bullet$	6–15	
Pileup	$\bullet$	6	
Simulation statistics	$\bullet$	Variable	
$\mathrm {DY}\,{\mathrm{Z}}\mathrm {jj}$ distribution (data)	$\bullet$	–	Variable
Theoretical			
PDF	$\bullet$	Variable	
$\mu _R/\mu _F$ (signal)	$\bullet$	Variable	
$\mathrm {DY}\,{\mathrm{Z}}\mathrm {jj}$ shape (MC)	$\bullet$	Variable	–
$\mathrm {DY}\,{\mathrm{Z}}\mathrm {jj}$ shape (PDF and $\mathrm {EW}\,\gamma \mathrm {jj}$ contribution)	$\bullet$	–	Variable
Interference	$\bullet$	100	
Normalisation of top-quark and diboson processes	$\circ$	7–10

**Table 3:** Event yields expected after fits to background and signal processes in methods A or B, using the initial selections (summarised in Table 1), and requiring ${S/B}>10\,\%$. The yields are compared to the data observed in the different channels and categories. The total uncertainties quoted for signal, $\mathrm {DY}\,{\mathrm{Z}}\mathrm {jj}$, dibosons (VV), and processes with top quarks (${\mathrm{t}}\overline{{\mathrm{t}}}$ and single top quarks) are dominated by JES uncertainties and include other sources, e.g., the statistical fluctuations in the MC samples

Selection	Channel	VV	Top quark	$\mathrm {DY}\,{\mathrm{Z}}\mathrm {jj}$	Total backgrounds	$\mathrm {EW}\,{\mathrm{Z}}\mathrm {jj}$	Data
Initial	$\mathrm {e}\mathrm {e}$ (A)	$255\,{\pm }\, 14$	$314\,{\pm }\, 15$	$20{,}083\,{\pm }\, 857$	${20{,}652\,{\pm } 857}\,$	$659\,{\pm }\, 16$	20,752
	$\mu \mu$ (A)	$355\,{\pm }\, 15$	$456\,{\pm }\, 16$	$30{,}042\,{\pm }\, 1{,}230$	${30{,}853 \,{\pm } \,1{,}230}$	$925\,{\pm }\, 22$	30,306
	$\mu \mu$ (B)	$226\,{\pm }\, 13$	$295\,{\pm }\, 12$	$25{,}505\,{\pm }\, 1{,}735$	${26{,}026 \,{\pm }\, 1{,}735}$	$833\,{\pm }\, 14$	26,651
BDT$>$0.05	$\mathrm {e}\mathrm {e}$ (A)	$56\,{\pm }\, 6$	$50\,{\pm }\,7$	$3{,}541\,{\pm }\, 169$	${3{,}647\,{\pm }\, 169}$	$427\,{\pm }\,12$	3,979
BDT$>$0.05	$\mu \mu$ (A)	$38\,{\pm }\, 5$	$36\,{\pm }\,5$	$2{,}867\,{\pm }\, 135$	${2{,}941 \,{\pm }\, 135}$	$459\,{\pm }\,14$	3,182
BDT$>$0.1	$\mu \mu$ (B)	$36\,{\pm }\, 3$	$35\,{\pm }\,3$	$3{,}871\,{\pm }\, 273$	${3{,}942 \,{\pm }\, 273}$	$514\,{\pm }\,12$	4,312

## Systematic uncertainties

The main systematic uncertainties affecting our measurement are classified into experimental and theoretical sources.

### Experimental uncertainties

The following experimental uncertainties are considered:

- Luminosity—A 2.6 % uncertainty is assigned to the value of the integrated luminosity [59].
- Trigger and selection efficiencies—We assign total 2 and 3 % uncertainties on the total trigger and selection efficiencies in the ee and $\mu \mu$ channels, respectively. These uncertainties have been estimated by comparing the lepton efficiencies expected in simulation and measured in data with a “tag-and-probe” method [60].
- Jet energy scale and resolution—The energy of the jets enters in our analysis not only at the selection level but also in the computation of the kinematic variables used in forming discriminants. The uncertainty on JES affects therefore both the expected event yields, through the migration of events to different bins, and the final distributions. In addition to the standard JES uncertainty, the residual difference in the response observed in the balancing of a ${\mathrm{Z}}$ or $\gamma$ candidate with a jet, discussed in Sect. 5, is assigned as a systematic uncertainty. The effect of the JES uncertainty is studied by rescaling up and down the reconstructed jet energy by a $p_{\mathrm {T}}$- and $\eta$-dependent scale factor [14]. An analogous approach is used for the JER. In both cases the uncertainties are derived separately of PF and JPT jets.
- q/g discriminator—The uncertainty on the performance of the q/g discriminator has been measured using independent ${\mathrm{Z}}$+jet and dijet data, after comparing with the corresponding simulation predictions [54]. The parametrization of the estimated uncertainty is used on an event-per-event basis to derive alternative predictions for the signal and background which are profiled in the fit for the signal.
- Pileup—Pileup is not expected to affect the identification and isolation of the leptons or the corrected energy of the jets. When the jet clustering algorithm is run, pileup can, however, induce a distortion of the reconstructed dijet system due to the contamination of tracks and calorimetric deposits. We evaluate this uncertainty by generating two alternative distributions after changing the number of pileup interactions by $\pm$5 %, according to the uncertainty on the inelastic pp cross section at $\sqrt{s}=8~\,\text {TeV}$.
- Statistics of simulation—For signal and backgrounds which are estimated from simulation we form envelopes for the distributions by shifting all bin contents simultaneously up or down by its statistical uncertainty. This generates two alternatives to the nominal shape to be analysed. However, when a bin has an uncertainty which is >$10\,\%$, we assign an additional, independent uncertainty to it in the fit in order to avoid overconstraining a specific background from a single bin in the fit.

**Table 4:** Event yields expected before the fit to background and signal processes in method C. The yields are compared to the data observed in the different channels and categories. The total systematic uncertainty assigned to the normalisation of the processes is shown

$M_\mathrm {jj}$ ($\text {GeV}$ )	Channel	VV	Top quark	$\mathrm {DY}\,{\mathrm{Z}}\mathrm {jj}$	Total backgrounds	$\mathrm {EW}\,{\mathrm{Z}}\mathrm {jj}$	Data
450–550	$\mathrm {e}\mathrm {e}$	$20\,{\pm }\,2$	$68\,{\pm }\,4$	$5{,}438\,{\pm }\,731$	${5{,}526\,{\pm }\,731}$	$94\,{\pm }\,6$	$5{,}809$
	$\mu \mu$	$27\,{\pm }\,2$	$96\,{\pm }\,4$	$7{,}325\,{\pm }\,983$	${7{,}448\,\,{\pm }\,983}$	$128\,{\pm }\,8$	$8{,}391$
550–750	$\mathrm {e}\mathrm {e}$	$16{\pm }1$	$56\,{\pm }\,3$	$3{,}802\,{\pm }\,496$	${3{,}874\,{\pm }\,664}$	$112\,{\pm }\,7$	$4{,}139$
	$\mu \mu$	$30\,{\pm }\,2$	$69\,{\pm }\,4$	$5{,}234\,{\pm }\,683$	${5{,}333\,{\pm }\,896}$	$155\,{\pm }\,10$	$5{,}652$
750–1,000	$\mathrm {e}\mathrm {e}$	$5.4\,{\pm }\,0.5$	$20\,{\pm }\,2$	$1{,}300\,{\pm }\,188$	${1{,}325{\pm }236}$	$73\,{\pm }\,5$	$1{,}384$
	$\mu \mu$	$7.5{\pm }0.6$	$26\,{\pm }\,2$	$1{,}846\,{\pm }\,262$	${1{,}880\,{\pm }\,313}$	$98\,{\pm }\,6$	$1{,}927$
$>$1,000	$\mathrm {e}\mathrm {e}$	$2.7\,{\pm }\,0.4$	$10.2\,{\pm }\,0.8$	$600\,{\pm }\,84$	${613\,{\pm }\,90}$	$84\,{\pm }\,6$	$684$
	$\mu \mu$	$4.2\,{\pm }\,0.4$	$13\,{\pm }\,1$	$913\,{\pm }\,127$	${930\,{\pm }\,122}$	$114\,{\pm }\,8$	$923$

**Table 5:** Fitted signal strengths in the different analyses and channels including the statistical and systematic uncertainties. For method C, only events with $M_\mathrm {jj}>450\,\text {GeV}$ are used. The breakup of the systematic components of the uncertainty is given in detail in the listings

	Analysis A			Analysis B	Analysis C		
	$\mathrm {e}\mathrm {e}$	$\mu \mu$	$\mathrm {e}\mathrm {e}+\mu \mu$	$\mu \mu$	$\mathrm {e}\mathrm {e}$	$\mu \mu$	$\mathrm {e}\mathrm {e}+\mu \mu$
Luminosity	0.03	0.03	0.03	0.03	0.03	0.03	0.03
Trigger/lepton selection	0.04	0.04	0.04	0.04	0.04	0.04	0.04
JES+residual response	0.06	0.05	0.05	0.04	0.06	0.05	0.05
JER	0.02	0.02	0.02	0.02	0.04	0.04	0.03
Pileup	0.01	0.02	0.02	0.01	0.01	0.01	0.01
$\mathrm {DY}\,{\mathrm{Z}}\mathrm {jj}$	0.07	0.05	0.07	0.08	0.14	0.12	0.13
q/g discriminator	$<$0.01	$<$0.01	$<$0.01	–	$<$0.01	$<$0.01	$<$0.01
Top, dibosons	0.01	0.01	0.01	0.01	$<$0.01	$<$0.01	$<$0.01
Signal acceptance	0.03	0.04	0.04	0.04	0.06	0.06	0.06
DY/EW ${\mathrm{Z}}$jj interference	0.14	0.14	0.14	0.13	0.06	0.08	0.08
Systematic uncertainty	0.19	0.18	0.19	0.17	0.17	0.17	0.18
Statistical uncertainty	0.11	0.10	0.07	0.09	0.24	0.21	0.16
$\mu =\sigma /\sigma _\text {th}$	0.82	0.86	0.84	0.89	0.91	0.85	0.88

### Theoretical uncertainties

We have considered the following theoretical uncertainties in the analysis:

- PDF—The PDF uncertainties are evaluated by considering the pdf4lhc prescription [18, 21–24], where for each source a new weight is extracted event-by-event and used to generate an alternative signal distribution. The up and down changes relative to the nominal prediction for each independent variable and are added in quadrature to estimate the final uncertainty.
- Factorisation and renormalisation scales—In contrast to the main background, the two signal process partons originate from electroweak vertices. Changing the QCD factorisation and renormalisation scales is therefore not expected to have a large impact on the final cross section. The renormalisation scale, in particular, is not expected to have any impact at LO. Changing the values of $\mu _F$ and $\mu _R$ from their defaults by 2 or 1/2 we find a variation of $\approx$ $4\,\%$ in MadGraph and in vbfnlo. As the change in the scales can also affect the expected kinematics, we use the altered $\mu _R/\mu _F$ samples to extract a weight that is applied at the generator level on an event-by-event basis. The parameterisation is done as function of the dilepton $p_{\mathrm {T}}$. The changes induced in the form of the discriminant at the reconstruction level are assigned as systematic uncertainties.
- DY Zjj prediction—For the modelling of the $\mathrm {DY}\,{\mathrm{Z}}\mathrm {jj}$ background from simulation, as we indicated previously, we consider the full difference between the Born-level MadGraph prediction and the NLO prediction based on mcfm as a systematic uncertainty. The differences are particularly noticeable at very large $M_\mathrm {jj}$ and at large $y^*$. For the data-based modelling of $\mathrm {DY}\,{\mathrm{Z}}\mathrm {jj}$ we consider the effect induced on the discriminant functions from five distinct sources. Not all are of theoretical nature, nevertheless, we list them here for simplicity. We consider not only the statistical size of the photon sample but also the difference observed in data selected with a loose-photon selection relative to the data selected with a tight-photon selection. From simulation, the expected difference, between the tight-photon selection and a pure photon sample is also considered, and added in quadrature to the previous. Furthermore, we consider the envelope of the PDF changes induced in the simulated compatibility tests, and the contamination from residual $\mathrm {EW}\,\gamma \mathrm {jj}$ events in the photon sample. For the latter, we assign a 30 % uncertainty to the $\mathrm {EW}\,\gamma \mathrm {jj}$ contribution, which is added in quadrature to the statistical uncertainty in the simulated events for this process.
- Normalisation of residual backgrounds—Diboson and top-quark processes are modelled with a MC simulation. Thus, we assign an intrinsic uncertainty in their normalisation according to their uncertainty which arises from the PDF and factorisation/renormalisation scales. The uncertainties are assigned based on [31, 37, 40].
- Interference between $\mathrm {EW}\,{\mathrm{Z}}\mathrm {jj}$ and $\mathrm {DY}\,{\mathrm{Z}}\mathrm {jj}$–The difference observed in the fit when the interference term is neglected relative to the nominal result is used to estimate the uncertainty due to the interference of the signal and the background.

### Summary of systematic uncertainties

Table 2 summarises the systematic uncertainties described above. We give their magnitudes at the input level, and whether they are treated as normalisation uncertainties or uncertainties in the distributions used to fit the data. The uncertainties are organised according to their experimental or theoretical nature.

## Measurement of the $\mathrm {EW}\,{\mathrm{Z}}\mathrm {jj}$ production cross section

Table 3 reports the expected and observed event yields after imposing a minimum value for the discriminators used in methods A and B such that ${S/B}>10\,\%$. Table 4 reports the event yields obtained in each category for method C. Fair agreement is observed between data and expectations for the sum of signal and background, for both methods, in all categories.

The signal strength is extracted from the fit to the discriminator shapes as discussed in Sect. 6. Table 5 summarises the results obtained for the fits to the signal strengths in each method. The results obtained are compatible among the dilepton channels and different methods, and in agreement with the SM prediction of unity. Methods A and B are dominated by the systematic uncertainty stemming from the modelling of the $\mathrm {DY}\,{\mathrm{Z}}\mathrm {jj}$ background and the interference with the $\mathrm {EW}\,{\mathrm{Z}}\mathrm {jj}$ signal. Method C is dominated by the statistical uncertainty in the fit and, due to tighter selection criteria, is expected to be less affected by the modelling of the interference. In method C, the $\mathrm {DY}\,{\mathrm{Z}}\mathrm {jj}$ modelling uncertainty is partially due to the statistics of the photon sample. With the exception of jet energy resolution, which has a larger impact in method C due to its tighter $M_\mathrm {jj}$ selection, all other uncertainties are of similar magnitude for the different methods.

**Fig. 10:**
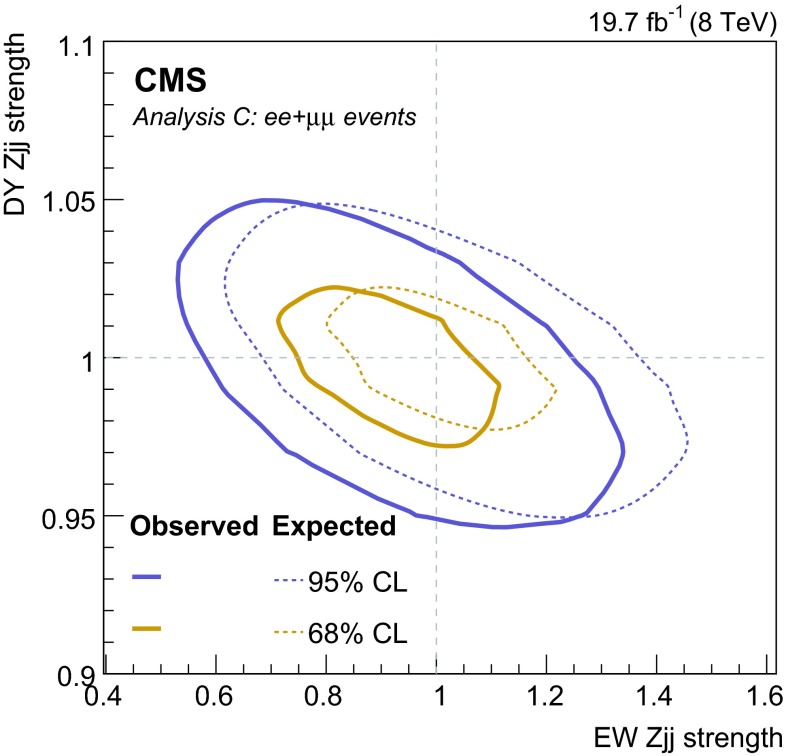
Expected and observed contours for the 68 and 95 % CL intervals on the $\mathrm {EW}\,{\mathrm{Z}}\mathrm {jj}$ and DY signal strengths, obtained with method C after combination of the ee and $\mu \mu$ channels

For the results from method C, the 68 and 95 % confidence levels (CL) obtained for the combined fit of the $\mathrm {EW}\,{\mathrm{Z}}\mathrm {jj}$ and $\mathrm {DY}\,{\mathrm{Z}}\mathrm {jj}$ strengths are shown in Fig. 10. Good agreement is found with the SM prediction for both components, as well as with the expected magnitude of the CL intervals. The $\mathrm {DY}\,{\mathrm{Z}}\mathrm {jj}$ strength is measured to be $0.978\pm 0.013\,\text {(stat)}\pm 0.036\,\text {(syst)}$ in the ee channel, $1.016\pm 0.011\,\text {(stat)}\pm 0.034\,\text {(syst)}$ in the $\mu \mu$ channel, and $0.996\pm 0.008\,\text {(stat)}\pm 0.025\,\text {(syst)}$ after the combination of the previous two.

From the combined fit of the two channels in analysis A we obtain the signal strength

$$\begin{aligned} \mu =0.84\pm 0.07\,\text {(stat)}\pm 0.19\,\text {(syst)}=0.84\pm 0.20\,\text {(total)}, \end{aligned}$$

corresponding to a measured signal cross section

$$\begin{aligned} \sigma ({\mathrm {EW}~\ell \ell \mathrm {jj}})&= &174\pm 15\,\text {(stat)}\pm 40\,\text {(syst)}\text {\,fb}\\&= &174\pm 42\,\text {(total)}\text {\,fb}, \end{aligned}$$

in agreement with the SM prediction $\sigma _\mathrm {LO}(\mathrm {EW}\,\ell \ell \mathrm {jj})=208\pm 18\text {\,fb}$. Using the same statistical methodology, as described in Sect. 6, the background-only hypothesis is excluded with a significance greater than 5$\sigma$.

**Fig. 11:**
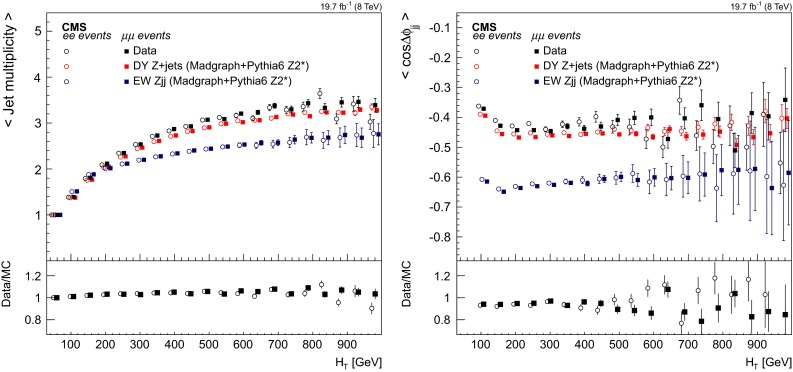
(*Left*) The average number of jets with $p_{\mathrm {T}} > 40\,\text {GeV}$ as a function of the total $H_{\mathrm {T}}$ in events containing a ${\mathrm{Z}}$ and at least one jet, and (*right*) average $\cos \Delta \phi _\mathrm {jj}$ as a function of the total $H_{\mathrm {T}}$ in events containing a ${\mathrm{Z}}$ and at least two jets. The ratios of data to expectation are given below the main panels. At each ordinate, the entries are separated for clarity. The expectations for $\mathrm {EW}\,{\mathrm{Z}}\mathrm {jj}$ are shown separately. The data and simulation points are shown with their statistical uncertainties

**Fig. 12:**
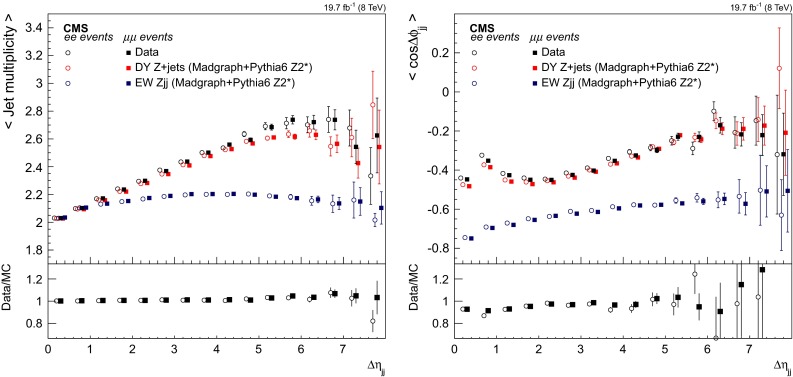
(*Left*) The average number of jets with $p_{\mathrm {T}} > 40\,\text {GeV}$ as a function of the pseudorapidity distance between the dijet with largest $\Delta \eta$, and (*right*) average $\cos \Delta \phi _\mathrm {jj}$ as a function of $\Delta \eta _\mathrm {jj}$ between the dijet with largest $\Delta \eta$. In both cases events containing a ${\mathrm{Z}}$ and at least two jets are used. The ratios of data to expectation are given below the main panels. At each ordinate, the entries are separated for clarity. The expectations for $\mathrm {EW}\,{\mathrm{Z}}\mathrm {jj}$ are shown separately. The data and simulation points are shown with their statistical uncertainties

## Study of the hadronic and jet activity in ${\mathrm{Z}}$+jet events

After establishing the signal, we examine the properties of the hadronic activity in the selected events. Radiation patterns and the profile of the charged hadronic activity as a function of several kinematic variables are explored in a region dominated by the main background, $\mathrm {DY}\,{\mathrm{Z}}\mathrm {jj}$; these studies are presented in Sects. 9.1 and 9.2. The production of additional jets in a region with a larger contribution of $\mathrm {EW}\,{\mathrm{Z}}\mathrm {jj}$ processes is furthermore pursued in Sect. 9.3. We expect a significant suppression of the hadronic activity in signal events because the final-state objects have origin in purely electroweak interactions, in contrast with the radiative QCD production of jets in $\mathrm {DY}\,{\mathrm{Z}}\mathrm {jj}$ events. The reconstructed distributions are compared directly to the prediction obtained with a full simulation of the CMS detector (see Sect. 3) and extends the studies reported in [61] to the phase space region of interest for the study of the $\mathrm {EW}\,{\mathrm{Z}}\mathrm {jj}$ process.

### Jet radiation patterns

For the ${\mathrm{Z}}$+jets events, the observables referred to as “radiation patterns” correspond to: (i) the number of jets, $N_\mathrm {j}$, (ii) the total scalar sum of the transverse momenta of jets reconstructed within $|\eta |<4.7$, $H_{\mathrm {T}}$, (iii) $\Delta \eta _\mathrm {jj}$ between the two jets with $p_{\mathrm {T}} >40\,\text {GeV}$ which span the largest pseudorapidity gap in the event (not required to be the two leading-$p_{\mathrm {T}}$ jets), and (iv) the cosine of the azimuthal angle difference, $\cos |\phi _{\mathrm {j}_1} - \phi _{\mathrm {j}_2} | = \cos \Delta \phi _\mathrm {jj}$, for the two jets with criterion (iii). These observables are measured using events that are required to satisfy the ${\mathrm{Z}}\rightarrow \mu \mu$ and ${\mathrm{Z}}\rightarrow \mathrm {e}\mathrm {e}$ selection criteria of analyses A and B. These observables are investigated following the prescriptions and suggestions from Ref. [62], where the model dependence is estimated by comparing different generators.

Figures 11 and 12 show the average number of jets and the average $\cos \Delta \phi _\mathrm {jj}$ as a function of the total $H_{\mathrm {T}}$ and $\Delta \eta _\mathrm {jj}$. The MadGraph + pythia (ME-PS) predictions are in good agreement with the data, even in the regions of largest $H_{\mathrm {T}}$ and $\Delta \eta _\mathrm {jj}$. In both cases we estimate that the contribution from $\mathrm {EW}\,{\mathrm{Z}}\mathrm {jj}$ is $<1\,\%$. Jet multiplicity increases both as function of $H_{\mathrm {T}}$ and $\Delta \eta _\mathrm {jj}$. The increase of $H_{\mathrm {T}}$ and $\Delta \eta _\mathrm {jj}$ induces, in average, an increase of jet multiplicity and leads to different dijet configurations in the azimuthal plane. In average the two selected jets are separated by $120^0\deg$, independently of $H_{\mathrm {T}}$. This separation tends to decrease for larger $\Delta \eta _\mathrm {jj}$ separation. The behavior observed for $\cos \Delta \phi _\mathrm {jj}$ when $\Delta \eta _\mathrm {jj}<0.5$ is related to the jet distance parameter used in the reconstruction (${R}=0.5$). In data, the separation of the jets in the $\cos \Delta \phi _\mathrm {jj}$ variable, is observed to be $<$5 % smaller with respect to the simulation.

**Fig. 13:**
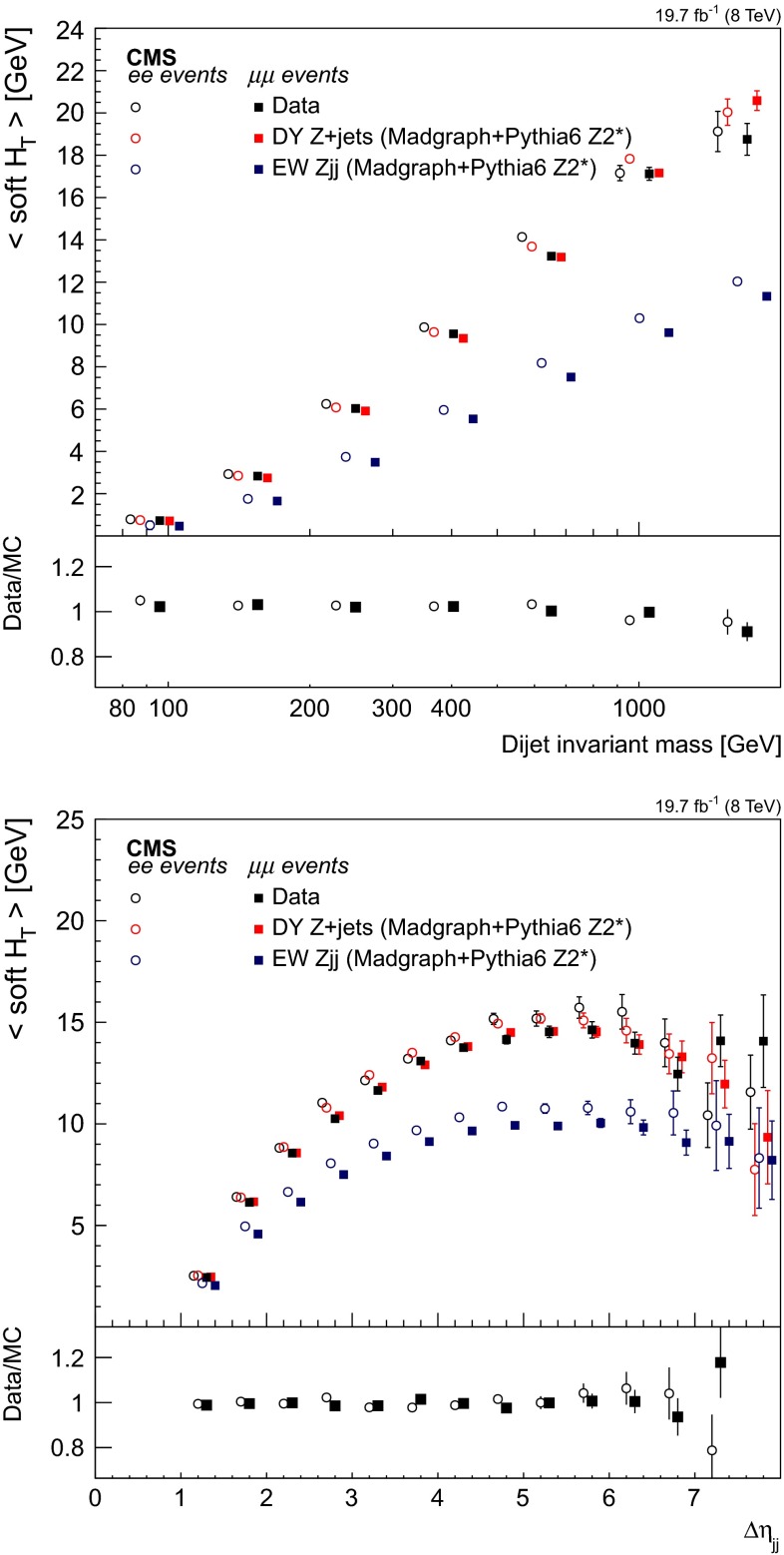
Average soft $H_{\mathrm {T}}$ computed using the three leading soft-track jets reconstructed in the $\Delta \eta _\mathrm {jj}$ pseudorapidity interval between the tagging jets that have $p_{\mathrm {T}} >50\,\text {GeV}$ and $p_{\mathrm {T}} >30\,\text {GeV}$. The average soft $H_{\mathrm {T}}$ is shown as function of: (*top*) $M_\mathrm {jj}$ and (*bottom*) $\Delta \eta _\mathrm {jj}$ for both the dielectron and dimuon channels. The ratios of data to expectation are given below the main panels. At each ordinate, the entries are separated for clarity. The expectations for $\mathrm {EW}\,{\mathrm{Z}}\mathrm {jj}$ are shown separately. The data and simulation points are shown with their statistical uncertainties

**Fig. 14:**
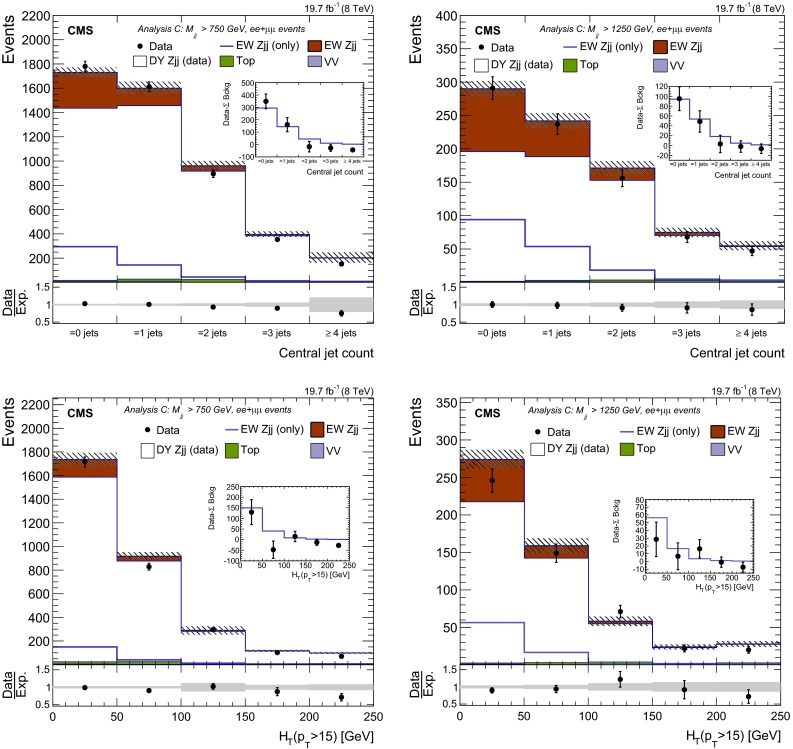
Additional jet multiplicity (*top row*), and corresponding $H_{\mathrm {T}}$ (*bottom row*) within the $\Delta \eta _{\mathrm {jj}}$ of the two tagging jets in events with $M_\mathrm {jj}>750\,\text {GeV}$ (*left column*) or $M_\mathrm {jj}>1,250\,\text {GeV}$ (*right column*). In the main panels the expected contributions from $\mathrm {EW}\,{\mathrm{Z}}\mathrm {jj}$, $\mathrm {DY}\,{\mathrm{Z}}\mathrm {jj}$, and residual backgrounds are shown stacked, and compared to the observed data. The signal-only contribution is superimposed separately and it is also compared to the residual data after the subtraction of the expected backgrounds in the *insets*. The ratio of data to expectation is represented by *point markers* in the *bottom panels*. The total uncertainties assigned to the expectations are represented as *shaded bands*

**Fig. 15:**
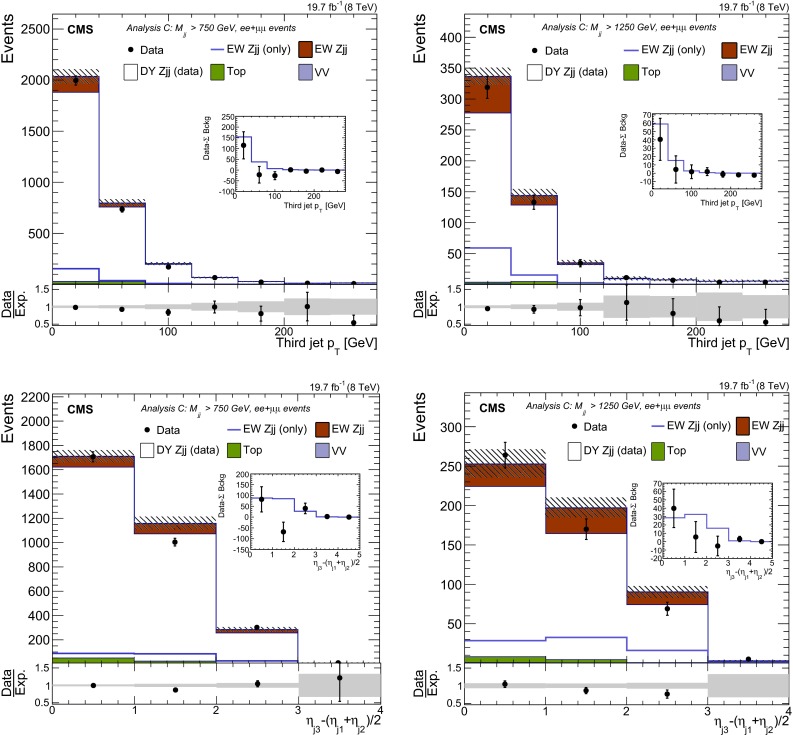
(*Top row*) $p_{\mathrm {T}}$ and (*bottom row*) $\eta ^*_{\mathrm {j}3}$ of the leading additional jet within the $\Delta \eta _{\mathrm {jj}}$ of the two tagging jets in events with $M_\mathrm {jj}>750\,\text {GeV}$ (*left column*) or $M_\mathrm {jj}>1{,}250\,\,\text {GeV}$ (*right column*). The explanation of the plots is similar to Fig. 14

**Fig. 16:**
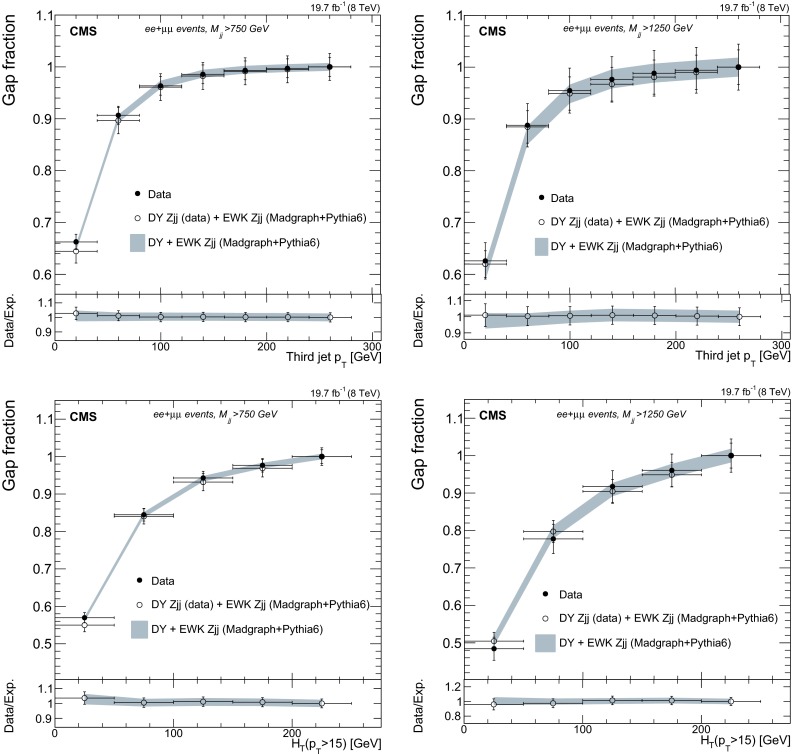
Gap fractions for: (*top row*) $p_{\mathrm {T}}$ of leading additional jet, (*bottom row*) the $H_{\mathrm {T}}$ variable within the $\Delta \eta _{\mathrm {jj}}$ of the two tagging jets in events with $M_\mathrm {jj}>750\,\text {GeV}$ (*left*) or $M_\mathrm {jj}>1{,}250\,\text {GeV}$ (*right*). The observed gap fractions in data are compared to two different signal plus background predictions where $\mathrm {DY}\,{\mathrm{Z}}\mathrm {jj}$ is modelled either from $\gamma \mathrm {jj}$ data or from simulation. The *bottom panels* show the ratio between the observed data and different predictions

### Study of the charged hadronic activity

For this study, a collection is formed of high-purity tracks [63] with $p_{\mathrm {T}} > 0.3\,\text {GeV}$, uniquely associated with the main PV in the event. Tracks associated with the two leptons or with the tagging jets are excluded from the selection. The association between the selected tracks and the reconstructed PVs is carried out by minimising the longitudinal impact parameter which is defined as the $z$-distance between the PV and the point of closest approach of the track helix to the PV, labeled $d_z^\mathrm {PV}$. The association is required to satisfy the conditions $d_z^\mathrm {PV}<2\text {\,mm}$ and $d_z^\mathrm {PV}<3\delta d_z^\mathrm {PV}$, where $\delta d_z^\mathrm {PV}$ is the uncertainty on $d_z^\mathrm {PV}$.

A collection of “soft track-jets” is defined by clustering the selected tracks using the anti-$k_{\mathrm {T}}$ clustering algorithm [47] with a distance parameter of $R=0.5$. The use of track jets represents a clean and well-understood method [64] to reconstruct jets with energy as low as a few $\text {GeV}$ . These jets are not affected by pileup, because of the association of their tracks with the hard-scattering vertex [65].

To study the central hadronic activity between the tagging jets, only track jets of low $p_{\mathrm {T}}$, and within $\eta ^\text {tag jet}_\text {min}+0.5 < \eta < \eta ^\text {tag jet}_\text {max}-0.5$ are considered. For each event, we compute the scalar sum of the $p_{\mathrm {T}}$ of up to three leading-$p_{\mathrm {T}}$ soft-track jets, and define it as the soft $H_{\mathrm {T}}$ variable. This variable is chosen to monitor the hadronic activity in the rapidity interval between the two jets.

The dependence of the average soft $H_{\mathrm {T}}$ for the ${\mathrm{Z}}\mathrm {jj}$ events as a function of $M_\mathrm {jj}$ and $\Delta \eta _\mathrm {jj}$ is shown in Fig. 13. Inclusively, the contribution from $\mathrm {EW}\,{\mathrm{Z}}\mathrm {jj}$ is estimated to be at the level of 1 %, but it is expected to evolve as function of the different variables, being 5 % (20 %) for $\vert \Delta \eta _\mathrm {jj}\vert >4$ ($M_\mathrm {jj}>1\,\text {TeV}$). Overall, good agreement is observed between data and the simulation. The average value of the soft $H_{\mathrm {T}}$ is observed to increase linearly with $M_\mathrm {jj}$, and to saturate its value for $\Delta \eta _\mathrm {jj}>5$, as a consequence of the limited acceptance of the CMS tracker.

### Jet activity studies in a high-purity region

The evidence for EW production of $\ell \ell \mathrm {jj}$ final states can also be supported through a study of the emission of a third and other extra jets in a region of high signal purity, i.e. for large $M_{jj}$. In this study, we compare two regions, one with $M_\mathrm {jj}>750\,\text {GeV}$ and another with $M_\mathrm {jj}>1{,}250\,\text {GeV}$. Aside from the two tagging jets used in the preselection, we use all PF-based jets with a $p_{\mathrm {T}} >15\,\text {GeV}$ found within the $\Delta \eta _\mathrm {jj}$ of the tagging jets. The background is modelled from the photon control sample (analysis C), and uses the normalisations obtained from the fit discussed in Sect. 8. Where relevant we also compare the results using the MC-based modelling of the background.

The number of extra jets, as well as their scalar $p_{\mathrm {T}}$ sum ($H_{\mathrm {T}}$), are shown in Fig. 14. Data and expectations are generally in good agreement for both distributions in the two $M_\mathrm {jj}$ regions. A clear suppression of the emission of a third jet is observed in data, when we take into account the background-only predictions. After subtraction of the background, which is shown as an inset in the different figures, we observe that slightly less extra jets tend to be counted in data with respect to the simulated signal. Notice that in the simulation of the signal, the extra jets have their origin in a parton-shower approach (see Sect. 3).

The $p_{\mathrm {T}}$ values and the pseudorapidities relative to the average of the two tagging jets, i.e. $\eta ^*_{\mathrm {j}3}=\eta _{\mathrm {j}3}-(\eta _{\mathrm {j}1}+\eta _{\mathrm {j}2})/2$, of the third leading-$p_{\mathrm {T}}$ jet in the event, are shown in Fig. 15. There are some deviations of the data observed relative to the predictions. In particular, the third jet is observed to be slightly more central than expected. The poor statistical and other uncertainties prevent us, however, from drawing further conclusions.

The above distributions can be used to compute gap fractions. We define a gap fraction as the fraction of events which do not have reconstructed kinematics above a given threshold. The most interesting gap fractions can be computed for the $p_{\mathrm {T}}$ of the leading additional jet, and the $H_{\mathrm {T}}$ variable. These gap fractions are, in practice, measurements of the efficiency of extra jet veto in VBF-like topologies. By comparing different expectations with the observed data we can quantify how reliable is the modelling of the extra jet activity, in particular in a signal-enriched region. Figure 16 shows the gap fractions expected and observed in data. Two expectations are compared: the one using a full MC approach and the one where the $\mathrm {DY}\,{\mathrm{Z}}\mathrm {jj}$ background is predicted from the $\gamma \mathrm {jj}$ data. Both predictions are found to be in agreement with the data for the $p_{\mathrm {T}}$ of the leading additional jet and the soft $H_{\mathrm {T}}$ variable.

## Summary

The cross section for the purely electroweak production of a Z boson in association with two jets in the $\ell \ell \mathrm {jj}$ final state, in proton–proton collisions at $\sqrt{s}=8\,\text {TeV}$ has been measured to be

$$\begin{aligned} \sigma ({\mathrm {EW}~\ell \ell \mathrm {jj}})=174\pm 15\,\text {(stat)}\pm 40\,\text {(syst)}\text {\,fb}, \end{aligned}$$

in agreement with the SM prediction. Aside from the two analyses previously used to determine the cross section of this process at 7$\,\text {TeV}$ [11], a new analysis has been implemented using a data-based model for the main background. The increased integrated luminosity recorded at 8$\,\text {TeV}$, an improved selection method, and more precise modelling of signal and background processes have allowed us to obtain a more precise measurement of the $\mathrm {EW}\,{\mathrm{Z}}\mathrm {jj}$ process relative to the 7$\,\text {TeV}$result.

Studies of the jet activity in the selected events show generally good agreement with the MadGraph +pythia predictions. In events with high signal purity, the additional hadron activity has also been characterised, as well as the gap fractions. Good agreement has been found between data and QCD predictions.
